# Genomic sequence context differentially impacts stepwise oxidation by TET enzymes to shape DNA hydroxymethylation landscapes

**DOI:** 10.1093/nar/gkag956

**Published:** 2026-10-13

**Authors:** Noa Erlitzki, Christian E Loo, Johanna M Fowler, Wanding Zhou, Rahul M Kohli

**Affiliations:** Graduate Group in Biochemistry, Biophysics, and Chemical Biology, Perelman School of Medicine, University of Pennsylvania, Philadelphia, PA 19104, United States; Department of Medicine, Perelman School of Medicine, University of Pennsylvania, Philadelphia, PA 19104, United States; Graduate Group in Biochemistry, Biophysics, and Chemical Biology, Perelman School of Medicine, University of Pennsylvania, Philadelphia, PA 19104, United States; Department of Medicine, Perelman School of Medicine, University of Pennsylvania, Philadelphia, PA 19104, United States; Center for Computational and Genomic Medicine, The Children’s Hospital of Philadelphia, Philadelphia, PA 19104, United States; Department of Pathology and Laboratory Medicine, University of Pennsylvania, Philadelphia, PA 19104, United States; Department of Medicine, Perelman School of Medicine, University of Pennsylvania, Philadelphia, PA 19104, United States

## Abstract

Ten-eleven translocation (TET) enzymes reverse gene silencing imposed by 5-methylcytosine (5mC) in mammalian genomes by catalyzing the stepwise oxidation of 5mC to 5-hydroxymethylcytosine (5hmC), 5-formylcytosine (5fC), and 5-carboxycytosine (5caC). 5hmC both acts as an independent epigenetic mark and promotes replication-dependent DNA demethylation by antagonizing maintenance DNA methylation. Oxidation of 5hmC to 5fC/5caC instead marks the committed step in replication-independent DNA demethylation, whereby 5fC/5caC are actively excised and reverted to unmodified cytosine. Although prior work indicates that TET-mediated oxidation is influenced by bases neighboring 5mC, these studies have been unable to resolve more than two of the relevant species—5mC, 5hmC, and 5fC/5caC—at the same time. As 5hmC is the key fulcrum in DNA demethylation, we established a high-throughput enzymology approach to probe these three cytosine states at base resolution simultaneously across hundreds of sites. We reveal distinct context dependencies for 5hmC generation versus decay, with flanking bases differentially impacting 5hmC persistence. Notably, we demonstrate these predictable preferences in duplexed DNA are lost in single-stranded DNA, mechanistically linking these features to TET-mediated base-flipping. Furthermore, analysis of genomic 5hmC patterns uncovers evidence of sequence context bias in cellular 5hmC accumulation, suggesting a role for TET-intrinsic preferences in shaping cellular epigenetic landscapes.

## Graphical Abstract

**Graphical Abstract:**
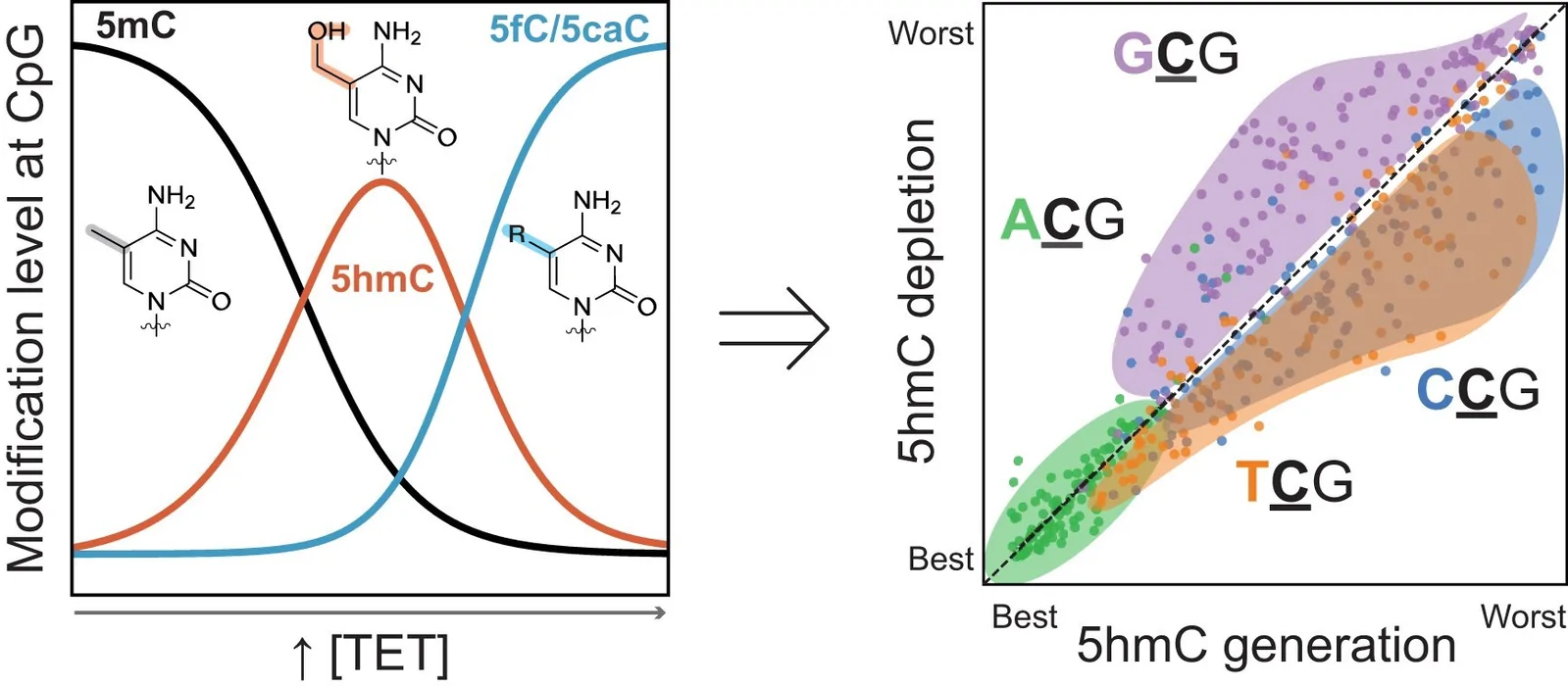

## Introduction

In mammalian genomes, 5-methylcytosine (5mC) occurs predominately in cytosine-guanine dinucleotide contexts (CpGs) and is best characterized for its prominent role in gene silencing [1]. Reversal of 5mC-associated gene silencing, known as DNA demethylation, can be driven by ten-eleven translocation (TET) family enzymes, which are Fe(II)/α-ketoglutarate (α-KG)-dependent dioxygenases [2]. TET enzymes catalyze the oxidation of 5mC to 5-hydroxymethylcytosine (5hmC), which can then be further oxidized to 5-formylcytosine (5fC) and 5-carboxycytosine (5caC) (Fig. 1) [3, 4]. As the most abundant of these oxidized 5mC bases, 5hmC is the dominant player in one of the established DNA demethylation pathways. In this replication-dependent pathway, poor recognition of 5hmC by the maintenance methyltransferase DNMT1 results in dilutional loss of modified cytosines [5]. A distinct replication-independent DNA demethylation pathway can also be mediated by 5fC/5caC, but not 5hmC. In this route, the highly oxidized TET products, 5fC/5caC, are substrates excised by thymine DNA glycosylase, with subsequent repair and replacement with unmodified cytosine completing the demethylation process [4, 6, 7]. As 5hmC is the substrate for 5fC/5caC generation, 5hmC thus exists as a key fulcrum along the TET oxidation pathways, separating replication-dependent and replication-independent DNA demethylation.

**Figure 1.**
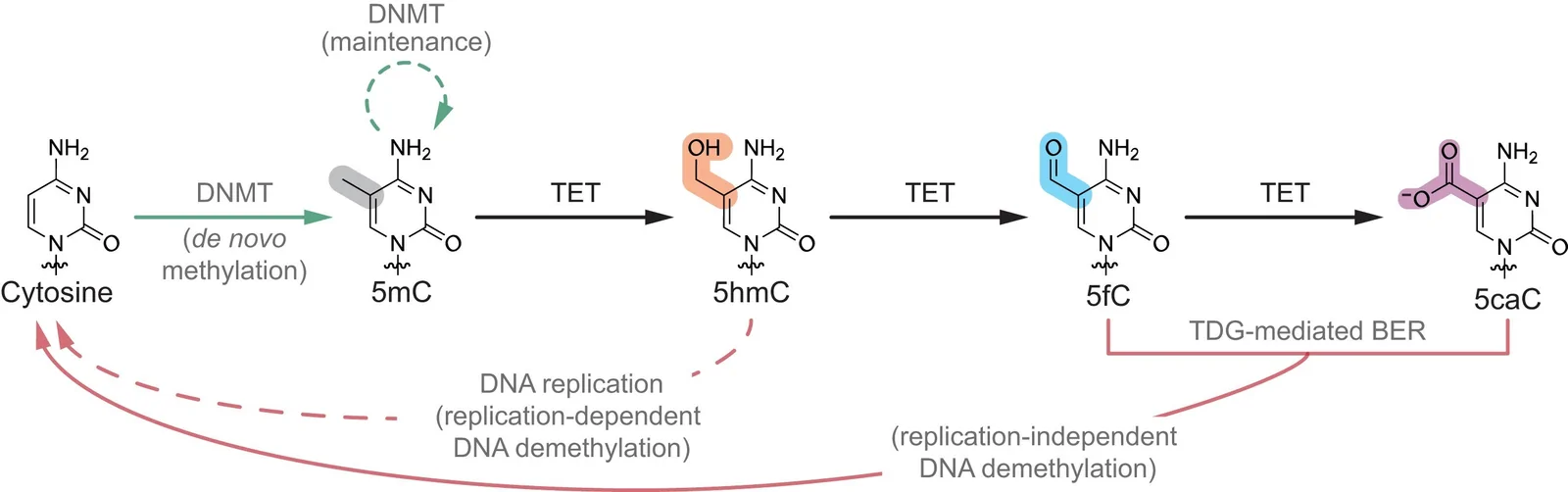
TET enzymes catalyze stepwise 5mC oxidation to 5hmC, 5fC, and 5caC, supporting distinct pathways for DNA demethylation.

In addition to serving as an intermediate for DNA demethylation, 5hmC also has independent roles as a *bona fide* epigenetic mark in and of itself. In contrast to 5fC/5caC, which are typically present in only 1 in 10^5^–10^6^ bases, 5hmC can constitute as much as 30% of the total modified cytosine bases in some cell types [8–10]. These 5hmC marks can be long-lived, with an abundance that varies reproducibly across different cell and tissue types [11–13]. Initial work building toward an atlas of 5hmC across tissues has highlighted that, much like unmodified CpGs in critical gene promoters, the landscape of 5hmC can define cell identity types [10, 11]. As a stable modification, 5hmC is most associated with gene activation, likely reflecting its effects on interactions between transcriptional machinery and DNA [10, 11, 14].

The dual roles of 5hmC as both an intermediate in DNA demethylation and as a stable epigenetic mark underscore the importance of better delineating the factors that dictate 5hmC turnover and accumulation. At the enzymatic level, these dynamics are determined by the balance between TET-mediated 5hmC generation from 5mC and 5hmC decay via further oxidation to 5fC. As the vast majority of 5mC is present in CpG dinucleotides, biochemical and structural studies have focused on understanding how TET enzymes engage with substrates in this context. Structural snapshots of TET2 have shown extensive engagement with the CpG dinucleotide, with the target cytosine base flipped out the DNA duplex and inserted into the active site [15, 16]. While the structures highlight distinct interactions with either 5mC, 5hmC, or 5fC as substrates, they also suggest minimal contacts with other neighbouring nucleobases flanking the CpG. Nonetheless, prior *in vitro* biochemical work has established that the genomic sequence context (i.e. the bases surrounding 5mCpGs) can significantly influence the overall efficiency of TET-mediated oxidation of 5mC to 5fC/5caC [17, 18]. Efforts to probe 5hmC dynamics, however, have been limited in their ability to discriminate the progression from 5mC to 5hmC and onward to 5fC/5caC, given technological constraints for localizing modifications (Supplementary Fig. S1). Taken together, these studies suggest a residual blind spot in understanding broader influences of sequence context on 5hmC dynamics.

An improved understanding of TET preferences for generating and depleting 5hmC *in vitro* could help better inform models for how TET enzymes reshape epigenomes in complex cellular settings. Here, building on recent precedents in “deep enzymology” approaches [17–24], we developed and employed a sequencing-based, high-throughput *in vitro* assay to parse the generation, depletion, and turnover of 5hmC at base resolution across hundreds of CpG sites within different sequence contexts. Our approach leverages a combination of chemical and enzymatic deamination steps that allow for quantification of 5mC, 5hmC, and 5fC/5caC in complex DNA substrates and reveals that sequence context can differentially impact 5hmC generation versus 5hmC decay. We show with multiple complex substrates that these attributes can be predicted in duplexed DNA, but are lost in single-stranded DNA (ssDNA), suggesting a potential mechanistic link between sequence preferences and the base-flipping mechanism involved in TET-mediated oxidation. Furthermore, we newly reexamine prior studies mapping 5hmC in cellular genomes and find evidence for the biased accumulation of 5hmC in preferred sequence contexts, providing new insights into TET-intrinsic features that can interact with genomic context to ultimately shape the landscape of 5hmC within cells.

## Materials and methods

### Preparation of CpG-methylated DNA substrates

#### Context-diverse methylated DNA substrate

A 4612-bp plasmid encoding the CpG-specific DNA methyltransferase M.MpeI was used to generate the 5mCpG-modified DNA substrate for assays (pMG81-MMpeI, referred to as pTW001). The plasmid, containing a total of 492 CpG sites, was introduced into chemically competent ER1821 *Escherichia coli*, a cell line that tolerates non-canonical DNA methylation [25]. With this system, leaky expression of M.MpeI results in self-methylation of the encoding plasmid at CpG sites. After overnight growth, the plasmid was extracted and purified using the QIAprep Spin Miniprep Kit (Qiagen). Although overall methylation was already high after leaky expression of the CpG-specific methyltransferase, the plasmid was further methylated *in vitro* using recombinant M.SssI (New England Biolabs (NEB)) per the manufacturer’s recommended protocol and then purified using the Zymo DNA Clean & Concentrator kit (Zymo Research). The final substrates were sequenced to >90× average depth and validated to be >97% CpG methylated (Supplementary File 2.A1 and Supplementary Fig. S2) using standard bisulfite sequencing (BS-Seq), as described below. The reference sequence for the context-diverse methylated DNA substrate is provided in Supplementary File 2.A2.

#### Methylated KvDMR-containing substrate

A 7119-bp plasmid with a total of 740 CpG sites was generated by subcloning the sequence of the murine KvDMR imprinting control region (ICR) (i.e. Kcnq1ot1; chr7:143294831–143296905, mm10) into the pMG81-MMpeI plasmid described earlier. The gene encompassing the KvDMR locus was obtained in two overlapping fragments (Twist Biosciences). The 5′-fragment contained overlap with original pMG81-MMpeI backbone 5′ to the insertion site (189 bp), followed 19 bp of the mm10 sequence immediately preceding the KvDMR locus, and then the first 1073 bp of the murine KvDMR locus. The 3′-fragment contained the remainder of the KvDMR locus, including overlap with the 5′-fragment (37 bp), the mm10 sequence immediately following the KvDMR locus (21 bp), and 187 bp of DNA overlapping the original pMG81-MMpeI backbone 3′ to the insertion site. The plasmid backbone fragment was generated by amplifying pMG81-MMpeI with overhangs to add homology. A three-piece Gibson ligation yielded the final plasmid containing the new KvDMR locus and still encoding the M.MpeI methyltransferase (pNE031). As with pMG81-MMpeI, the plasmid was transformed into chemically competent ER1821 *E. coli* and grown overnight to allow for leaky expression of M.MpeI and subsequent self-methylation at CpGs, with the extracted and purified plasmid then further methylated *in vitro* with M.SssI, sequenced to >70× average depth, and validated to be >97% CpG methylated by BS-Seq (Supplementary File 2.A3 and Supplementary Fig. S2). The reference sequence for methylated KvDMR-containing substrate is provided in Supplementary File 2.A4.

#### 5mC-containing dsDNA, ssDNA, and mmDNA oligo substrates

Matched double-stranded DNA (dsDNA), ssDNA, and mismatch-DNA (mmDNA) substrates containing 5mC in known sequence contexts were used to study the mechanisms involved in defining sequence context preferences. For these assays, an 88-nt DNA oligonucleotide substrate (top88-1) was designed with a 5′-unique molecular identifier (UMI) marked by a D_12_ sequence (D = A, G, T) and containing four 5mCpGs sites within palindromic GCGC, ACGT, TCGA, and CCGG sequence contexts (IDT). This ssDNA oligo was used as is and designated “ssDNA-1” in assays requiring a single-stranded substrate. To make a dsDNA encoding the UMI (dsDNA-1), an unmethylated 76-nt oligo (bottom76-1) complementary to the non-UMI portion of top88-1 (IDT) was annealed and extended using Klenow Fragment (exo-) (NEB) per the recommended protocol and purified over Zymo Oligo Clean & Concentrator columns (Zymo Research). A separate dsDNA containing a 5mC:T mismatch at each CpG site (mmDNA-1) was likewise made using a mismatched analog of bottom76-1 (mismatch76-1). To evaluate another single-stranded substrate (ssDNA-2), a second 88-nt oligonucleotide (top88-2) was also employed, in which the order of the 5mCpGs in different sequence contexts was reversed, along with its corresponding fully matched (bottom76-2) or mismatched complement (mismatch76-2) to allow for generation of its dsDNA (dsDNA-2) and mmDNA analogs (mmDNA-2). To verify appropriate duplexing of dsDNA and mmDNA substrates, 25 ng of each top strand, bottom strand, and duplexed substrates were visualized on a native polyacrylamide gel electrophoresis (12% acrylamide/bis 19:1 in Tris-Borate-EDTA (TBE)) that was post-stained with SYBR Gold and imaged on a BioRad Molecular Imager Gel Doc XR+ (Supplementary Fig. S9). Sequences for all substrate oligonucleotides are provided in Supplementary File 2.A5.

### TET enzyme expression and purification

The crystallized construct of human TET2 (TET2-CS) [15] (which includes residues 1129−1936 and a Gly-Ser linker in place of residues 1481−1843) was subcloned into a pFastBac1 vector [26, 27]. Expression was carried out in Sf9 insect cells with purification using the N-terminal FLAG tag as previously described [28, 29]. Purified protein was stored in aliquots at −80°C to minimize exposure to freeze-thaw cycles.

### *In vitro* TET oxidation assays

#### Validation and selection of pyrC adapters

To explore the compatibility of various adapters with chemical and enzymatic deamination sequencing pipelines, a comparison was made between adapters containing unmodified cytosine, 5mC, 5-propynylcytosine (5pyC), and pyrrolocytosine (pyrC). Adapters containing C, 5pyC, or pyrC were synthesized by IDT, with sequences provided in Supplementary File 2.A5. 5mC-containing adapters were obtained from the NEBNext EM-Seq kit (NEB). The context-diverse methylated DNA plasmid was sheared to ~350 bp (Covaris) and end-repaired using the NEBNext Ultra II DNA Library Prep Kit (NEB). The 5′-phosphorylated, 3′-dA-tailed dsDNA fragments were ligated to the adapters containing either C, 5mC, 5pyC, or pyrC using the NEBNext Ultra II DNA Library Prep Kit (NEB), and the DNA was purified with a 1.2× left-sided SPRI purification (Beckman Coulter). From the resulting DNA, 21.5-μl solutions containing 100 ng of adapter-ligated DNA in nuclease-free water were prepared, of which 1.5 μl was reserved as untreated control and polymerase chain reaction (PCR) amplified as described the dual-sequencing pipeline below for a total of 12 cycles. Of the remaining 20 μl, half was treated with bisulfite only (BS) and half with bisulfite and A3A (bACE), as described in the dual-sequencing pipeline below. As with the untreated control, the post-BS and post-bACE samples were then amplified for 20 cycles and 22 cycles, respectively. The resulting amplification products were quantified using the Qubit dsDNA HS kit (Invitrogen) to determine whether library generation, which relies on the integrity of primer-binding sites within the adapters, was successful as an indication of compatibility of the tested modified adapters.

#### Assays with context-diverse substrates

80 nanograms of the appropriate DNA substrate (context-diverse methylated substrate or methylated KvDMR-containing substrate) was mixed with 0.4 ng (0.5% w/w) of an unmethylated phiX DNA (Thermo Fisher Scientific) as an internal spike-in control to validate conversion efficiency. The DNA mixture was sheared to ~350 bp (Covaris) and end-repaired using the NEBNext Ultra II DNA Library Prep Kit (NEB). The 5′-phosphorylated, 3′-dA-tailed dsDNA fragments were ligated to custom stubby adapters (IDT) containing pyrC in lieu of unmodified cytosine. The ligation was carried out using the NEBNext Ultra II DNA Library Prep Kit (NEB), and the DNA was purified with a 1.2× left-sided SPRI purification (Beckman Coulter). For the TET reaction, the adapter-ligated DNA was treated with 0–1.6 µM of TET in 1× TET reaction buffer (50 mM HEPES, 100 mM NaCl, 1 mM ascorbic acid, 1 mM a-ketoglutarate, pH 6.5). Reactions were initiated by the addition of ammonium iron(II) sulfate to a final concentration of 75 µM and allowed to proceed for 1 h at 37°C. Reactions were quenched by addition of a pre-mixed quenching solution comprised of two volume-equivalents of Oligo Binding Buffer (Zymo) and eight volume-equivalents of ethanol, followed by purification of DNA products over Zymo Oligo Clean & Concentrate columns (Zymo). TET-treated DNA was then taken through the dual-sequencing pipeline described below to evaluate the fraction of 5mC, 5hmC, and 5fC/5caC at each TET concentration at base resolution. Assays were performed in three independent replicates. With each replicate, rather than employing the same TET2-CS concentrations, the concentrations were staggered across the dynamic range to maximize sampling of points that define the dose-response curve. Data from the independent replicates were concatenated for fitting to a model of stepwise oxidation, described below, to extract TET activity parameters.

#### TET assays with 5mC-containing dsDNA, ssDNA, or mmDNA oligo substrates

5mC-containing dsDNA, ssDNA, and mmDNA oligo substrates were generated as described earlier. Using either 25 ng of ssDNA substrate or 50 ng of dsDNA substrate, reactions were carried out in 1× TET reaction buffer supplemented with 75 µM ammonium iron(II) sulfate, with reactions initiated by the addition of TET2-CS to a final concentration of 0.5 µM. Following incubation for 1 h at 37°C, reactions were quenched as described for context-diverse methylated substrate and purified over Zymo Oligo Clean & Concentrate columns (Zymo Research). Reactions were performed in triplicate, alongside a negative control (no TET) for each condition. For ssDNA samples, conversion to dsDNA was performed after TET reaction. The TET-reacted ssDNA molecules were annealed to their unmethylated complement strands, extended using Klenow Fragment (exo-), and purified over Zymo Oligo Clean & Concentrator columns as described earlier. All samples were then further processed as described for the context-diverse methylated substrate, including end-repair, ligation to custom pyrC-containing adapters (IDT), and processing through the dual-sequencing pipeline detailed below.

#### Time-resolved assays with the context-diverse methylated substrate

For comparison of results obtained from concentration-dependent titration of TET2-CS to time-dependent product formation, assays were additionally performed at a fixed TET concentration across serial time points. For time-resolved assays, 80 ng of pMG81-MMpeI or pNE031 was mixed with 0.4 ng (0.5% w/w) of an unmethylated phiX spike-in control (Thermo). The pooled DNA was sheared and end-repaired as above and then combined with 1.1 µM TET in 1× TET reaction buffer. Reactions were initiated by the addition of ammonium iron(II) sulfate to a final concentration of 75 µM and allowed to proceed at 37°C for various time points (*t* = 0, 0.5, 2, 3, 8, 20, 22, 60, or 65 min). After incubation, reactions were quenched by the addition of 500 mM ethylenediaminetetraacetic acid (EDTA) to a final concentration of 45 mM and stored at −20°C. For the *t* = 0 time point only, the reaction was pre-quenched by the inclusion of EDTA prior to reaction initiation, and then immediately frozen at −20°C after addition of ammonium iron(II) sulfate. Quenched reactions were purified over Zymo Oligo Clean & Concentrate columns, adapter-ligated, end-repaired, and purified with a 1.2× left-sided SPRI purification as described earlier, with the site-specific fraction of 5mC, 5hmC, and 5fC/5caC at each time point evaluated using the dual-sequencing pipeline described below. Time-resolved assays were performed in two independent replicates, with time points staggered between replicate assays to maximize coverage of the time range.

### Dual-sequencing pipeline for base-resolution identification of 5mC, 5hmC, and 5fC/5caC

DNA products described earlier were analyzed using a dual-sequencing pipeline. The pipeline employs BS-Seq to resolve 5fC/5caC from 5mC/5hmC, and bisulfite-assisted APOBEC-coupled epigenetic sequencing (bACE-Seq) to resolve 5mC from 5hmC, while also allowing for correction for the low background of unmodified C (see below). In this dual pipeline approach, DNA products were first processed using the Premium Bisulfite Kit (Diagenode) according to the manufacturer’s suggested protocol, except that the column-bound, bisulfite-treated DNA was subjected to an additional centrifugation step prior to elution in 20 μl of nuclease-free water. The resulting bisulfite-treated DNA was split into two 10-μl aliquots. One aliquot, designated the BS-Seq arm of the dual pipeline, was brought up to a final volume of 22.5 μl with nuclease-free water and stored at −20°C until a later indexing PCR step. The other 10 μl aliquot was designated for bACE-Seq [30–33] to facilitate enzymatic deamination of 5mC. In the bACE-Seq pipeline, the 10 μl of post-bisulfite treated DNA was denatured by the addition of 4 μl fresh formamide and brought to a final volume of 20 μl in nuclease-free water, followed by incubation at 85°C for 10 min. Immediately following denaturation, the samples were snap-cooled by transfer to a metal sample block pre-cooled at −20°C. After 1 min, the samples were incubated with APOBEC3A (A3A) in APOBEC Reaction Buffer supplemented with bovine serum albumin per the NEBNext Enzymatic Methyl-seq kit protocol (NEB). Samples were incubated at 37°C for 3 h, then subjected to 1.2× left-sided SPRI purification and eluted in 22.5 μl of nuclease-free water. The BS-Seq and bACE-Seq samples were then processed in parallel to introduce unique dual-index primer pairs via indexing PCR following the protocol from the NEBNext Multiplex Oligos for Enzymatic Methyl-seq kit (NEB). A final 0.9× (or 1.2× for oligo-based assays) left-sided SPRI purification was performed on the indexed samples. The resulting libraries were quantified using the Qubit dsDNA HS kit (Invitrogen), assessed for quality on either an Agilent BioAnalyzer or TapeStation instrument (Agilent), and pooled and sequenced on an Illumina MiSeq using 150-bp read lengths with either a Nano or Micro v2 300 cycle kit (Illumina). Raw (FASTQ files) and processed sequencing data (Bismark coverage files) are accessible at the Gene Expression Omnibus (GEO) under accession number GSE319425.

### Bioinformatic processing and analysis of epigenetic sequencing data

#### Processing of raw sequencing data

All data were processed in single-end mode using read 1 (R1). For assays using either the context-diverse methylated substrate or the methylated KvDMR-containing substrate, low-quality bases and adapter sequences were trimmed from raw reads using Trim Galore! (version 0.6.10) (https://github.com/FelixKrueger/TrimGalore), and trimmed reads with a length <20 bases were discarded. Bismark (version 0.23.1) (https://github.com/FelixKrueger/Bismark) was used to map trimmed reads to bisulfite-converted reference sequences prepared with bismark_genome_preparation. Mapped reads were sorted and indexed using SAMtools (1.6) and deduplicated with Bismark. The deduplicated, aligned reads were then filtered to account for A3A or bisulfite failure by removing reads with three or more consecutive non-converted CpH-context cytosines using the filter_non_conversion script in Bismark. Reads with MAPQ ≥ 30 were retained, and methylation status at each CpG was obtained using bismark_methylation_extractor. Downstream analyses were restricted to CpG sites with at least 10× coverage from any given sequencing run.

Processing of sequencing data from assays using 5mC-containing ssDNA or dsDNA oligos as substrates was similar, with the following exceptions. Raw reads were first split by top/bottom strand using motif-based detection, and bottom-strand reads were filtered out to focus analysis on the strand containing 5mC. Cutadapt (version 5.0) was used to trim adapter sequences only and discard trimmed reads shorter than 20 bases. Strand-specific reads were aligned to bisulfite-prepared reference strands using Bismark. UMIs from each read were propagated into RX tags (“RX:Z:<UMI>”) and used for deduplication with umi_tools (version 1.1.4). For removal of reads with excessive non-conversion, a filter_non_conversion threshold of two consecutive non-converted CpH sites was used. Finally, three read retention criteria were required for analysis: MAPQ ≥ 30, length exactly 76 nt, and base identities of either C or T at positions 15, 31, 47, and 63 maintained for subsequent extraction of CpG methylation status.

#### Calculation of 5mC, 5hmC, and 5fC/5caC levels

Parallel BS-Seq and bACE-Seq readouts were analyzed to resolve 5mC, 5hmC, and 5fC/5caC status at each site. For each CpG-context cytosine analyzed by BS-Seq, the number of “C” reads as 5mC/5hmC (denoted C_BS_) and the number of “T” reads as 5fC/5caC (denoted T_BS_) were counted. For bACE-Seq, the number of “C” reads as 5hmC (denoted C_bACE_) and the number of “T” reads as 5mC/5fC/5caC (denoted T_bACE_) were counted. The “true 5hmC” level was given by C_bACE_, and the “true 5fC/5caC” level was given by T_BS_. The “true 5mC” level was obtained by subtracting C_bACE_ from C_BS_. At each CpG site, to account for non-methylated CpGs in the starting material or non-conversion of 5mC by A3A, the baseline methylation level and false detection rate (FDR) of 5hmC were determined, respectively, from the BS-Seq and the bACE-Seq data in the absence of TET. Baseline-adjusted values of 5mC, 5hmC, and 5fC/5caC as fractions of total modified cytosine (denoted frac_mC, frac_hmC, and frac_fCcaC, respectively) were calculated for each CpG site. CpG positions for which the FDR exceeded 0.10 or with fewer than 10 reads in either the BS-Seq or the bACE-Seq data were excluded from downstream analysis (Supplementary Fig. S3).

#### Extraction of TET activity parameters from TET concentration-dependent activity assays

Levels of 5mC, 5hmC, and 5fC/5caC (as fractions of total modified cytosine, calculated as described earlier) were concatenated from replicate experiments and plotted as a function of TET concentration. Data points with values of fraction modified 5mC (frac_mC), 5hmC (frac_hmC), or 5fC/5caC (frac_fCcaC) less than −0.05 or >1.05 were excluded. Linked constants (EC50_1_ and EC50_2_) were obtained by fitting the data to a nine-parameter system of Hill-like equations modeling 5hmC turnover as the combined outcome of both 5mC depletion (from an initial maximum of 100% 5mC) and 5fC/5caC generation (up to a final maximum of 100% 5fC/5caC):


(1) \begin{eqnarray*}{\mathrm{frac\_mC}} = {\mathrm{\ }}\frac{1}{{1 + {{{\left( {\frac{{EC{{{50}}_1}}}{{\left[ {{\mathrm{TET}}} \right]}}} \right)}}^{{{n}_1}}}}},\end{eqnarray*}

(2) \begin{eqnarray*}{\mathrm{frac\_hmC}} = {\mathrm{\ }}\mathrm{ pea}{{\mathrm{ k}}_h} - \frac{1}{{1 + {{{\left( {\frac{{EC{{{50}}_{12}}}}{{\left[ {{\mathrm{TET}}} \right]}}} \right)}}^{{{n}_{12}}}}}} - \frac{1}{{1 + {{{\left( {\frac{{EC{{{50}}_{21}}}}{{\left[ {{\mathrm{TET}}} \right]}}} \right)}}^{{{n}_{21}}}}}},\end{eqnarray*}

(3) \begin{eqnarray*}{\mathrm{frac\_fCcaC}} = \ \frac{1}{{1 + {{{\left( {\frac{{EC{{{50}}_2}}}{{\left[ {{\mathrm{TET}}} \right]}}} \right)}}^{{{n}_2}}}}}.\end{eqnarray*}

The primary parameters of interest (POIs) are EC50_1_ and EC50_2_. The efficiency of 5hmC generation (corresponding to the 5mC → 5hmC transition) is approximated by EC50_1_ (the concentration of TET at which half of all 5mC sites were oxidized to 5hmC). Similarly, the efficiency of 5hmC depletion (corresponding to the 5hmC → 5fC/5caC transition) is approximated by EC50_2_ (the concentration of TET at which half of all 5mC sites were further oxidized from 5hmC to 5fC/5caC). EC50_12_ and EC50_21_ describe the apparent efficiencies of net accumulation and net loss of 5hmC, reaching a maximum at peak_h_ (the TET concentration at which the gain and loss of 5hmC are in macroscopic equilibrium), that result from the coupled dynamics of 5hmC generation and depletion. As these two parameters absorb heterogeneity from the aggregate contributions of multiple molecular transitions and populations, they are critical to the overall fit of the data but are less useful for downstream analysis than EC50_1_ and EC50_2_, which provide unconfounded measures of 5hmC generation and depletion efficiencies, respectively. Hill slope-like exponents (*n*_1_, *n*_12_, *n*_21_, and *n*_2_) are included only as phenomenological parameters to capture the steepness of each transition; no specific biophysical interpretation is ascribed to their values.

Parameters were estimated by nonlinear least-squares using the SciPy least_squares optimizer to simultaneously fit the data to the system of equations. Standard errors were determined based on estimated parameter covariance computed from the solver’s Jacobian. The optimizer was initialized at [EC50_1_, n_1_, peak_h_, EC50_12_, *n*_12_, EC50_21_, *n*_21_, EC50_2_, *n*_2_] = [0.05, −2, 0.6, 0.03, −1, 0.35, 1, 0.3, 2] and loosely constrained with the following bounds:

EC50_1_, EC50_12_, EC50_21_, EC50_2_ ∈ [10^− 6^, 5] μM

*n*_1_, *n*_12_ ≤ 0; *n*_2_, *n*_21_ ≥ 0

peak_h_ ≥ 0

As frac_mC is derived by subtraction, it inherits correlated error from both BS-Seq and bACE-Seq measurements. To maintain clean error structure in the extracted fitting parameters, we only explicitly passed through the optimizer the directly measured observables: frac_fC, frac_hmC, and the two composite series frac_mC + hmC (corresponding to C_BS_) and frac_mC + fCcaC (corresponding to T_bACE_). Simultaneous fitting of these observables implicitly defines frac_5mC and inherently enforces unity (ensuring that frac_mC + frac_hmC + frac_fCcaC = 1).

Fitted parameters were inspected to identify unreliable parameter estimates due to poor fits to the data, insufficient coverage across the dynamic range, or collinearity between POIs. To aid inspection of parameters extracted from fitting of individual CpG sites, CpGs were flagged if (i) there were fewer than 10 total data points representing unique TET concentrations across the dynamic range (those with fewer than 5 were automatically excluded from fitting and further analysis), (ii) goodness-of-fit analysis had an adjusted-*R*^2^ value <0.85, or (iii) there was evidence of potential collinearity or instability among any of the nine parameters, defined as a Gauss–Newton condition number of κ(J⊤J) > 10^10^. CpG sites with data for which the fit yielded high relative standard errors in POIs (RSE(EC50_1_) > 0.8 or RSE(EC50_2_) > 0.5) or high collinearity between POIs (from the covariance matrix, absolute correlation between EC50_1_ and EC50_2_ > 0.9) were deemed “POI-unstable” and excluded from further downstream analysis. For fitting of aggregate data to estimate global TET activity parameters, each data point was the mean ± SE of the value across all CpG sites in the DNA substrate (excluding any for which the individual fit to the data returned unstable POIs), and the residuals were weighted by the SEs. A flow diagram showing attrition through the analysis pipeline of data from titration experiments utilizing the pMG81-MMpeI substrate is shown in Supplementary Fig. S3.

#### Analysis of sequence context-specific 5modC and 5hmC levels in mouse neurons and ESCs

To investigate sequence context bias in genomic 5hmC patterns, we analyzed publicly available whole-genome sequencing datasets from the GEO focusing on two cell types: mouse excitatory neuronal cells and embryonic stem cells (ESCs). The analysis incorporated datasets that profile 5modC (5mC/5hmC together) using standard BS-Seq, as well as datasets that specifically profile 5hmC using either TET-assisted bisulfite sequencing (TAB-Seq) or ACE-Seq methodologies (GEO accession numbers: GSM1173794, GSM1173795, GSM1180306, GSM1180307, GSM1180308, GSM3207181, GSM3207182, GSM3207185, GSM3207186) [30, 34–36]. Raw sequencing data were processed using the default Bismark-based Snakemake pipeline with strand-specific output, retaining individual cytosine information rather than merging palindromic cytosines by CpG pairs.

To assess sequence context bias, we focused on 4-mer sequence contexts centered on CpG dinucleotides, specifically examining the effect of the −1 (N in NCG) and +2 (N in CGN) positions relative to the CpG. Methylation data aggregation across different 4-mer classes was performed using the YAME tool [37] with the command “yame summary -m ChromHMM_kmer4.20240219.cm” to generate summary statistics for modification levels stratified by sequence context. The 4-mer reference knowledge base was created by tallying all sequence contexts in the GRCm38/mm10 reference genome. For each cell type and sequencing modality, we calculated the frequency of modification with which modified CpGs (5modC or 5hmC) observed on each CpG then averaged by the 4-mer context. The 4-mer context is further aggregated into CpG’s NCG or CGN sequence context, normalized by the genome-wide prevalence of each respective 3-mer sequence motif. This normalization approach allowed us to identify enrichment or depletion of modifications in specific sequence contexts relative to their genomic baseline.

#### Analysis of 5hmC sequence context at transcription factor binding sites

Genome-wide 5hmC maps for mouse neurons and ESCs were obtained from published ACE-Seq datasets and reprocessed uniformly [30]. Reads were aligned to the mm10 genome with Bismark, and 5hmC levels were quantified at strand-specific CpG resolution. To relate 5hmC to the sequence context recognized by transcription factors (TFs), predicted TF binding sites for mm10 were downloaded from the JASPAR 2024 database via the UCSC Genome Browser and separated by motif [38]. For each TF, CpG sites overlapping its binding sites were identified by intersection, and each overlapping CpG was annotated with the base identity at the −1 and +2 flanking positions. The base composition frequencies at the −1 and +2 positions were tabulated for each TF, retaining TFs with at least 500 000 overlapping CpG observations. The resulting frequency matrix, with features defined by base identity at each flanking position, was scaled and subjected to principal component analysis. Each TF was projected onto the first two principal components, with the loadings of the flanking-position features overlaid as vectors. The aggregate 5hmC level across the CpGs overlapping each TF was computed from the ACE-Seq data and used to color the projection.

### Data visualization and statistical analyses

#### Data visualization

Plots were generated using matplotlib (version 3.10.6) via a custom Python script or in Prism 10 version 10.3.1 for MacOS (GraphPad Software, Boston, Massachusetts USA, www.graphpad.com). DNA sequence logos were generated with logomaker (version 0.8) (https://github.com/jbkinney/logomaker) using per-position probabilities calculated from base counts.

#### Statistical methods

Statistical analyses of data obtained in this study were performed in Python (version 3.11.13) using statsmodels (version 0.14.5) and pingouin (version 0.5.5) in custom scripts. Group comparisons of continuous parameters (e.g. EC50_1_ values) were performed using Welch’s analysis of variance (ANOVA), followed by Games–Howell post hoc testing for pairwise comparisons. Group comparisons of ranked parameters (e.g. quantile EC50_1_ values) were performed using the Kruskal–Wallis test across groups, followed by pairwise Mann–Whitney U testing with Holm–Bonferroni correction for multiple comparisons. Statistical testing for analysis of mouse 5modC and 5hmC data downloaded from the GEO repository was performed in Prism 10 version 10.3.1 for MacOS (GraphPad Software, Boston, Massachusetts USA, www.graphpad.com) using repeated measures one-way ANOVA with Tukey’s multiple comparisons test.

### Custom scripts and programs

Scripts used for data processing, fitting, and analysis are freely available at https://github.com/erlitzkin/TET-seq-context-prefs. AI (ChatGPT, OpenAI) was used to assist with refining code, debugging, and editing of custom analysis scripts.

## Results

### High-throughput, base-resolution profiling of 5mC oxidation products

To evaluate the dynamics of 5hmC generation and decay *in vitro*, we designed a sequencing-based assay that allowed us to simultaneously track 5mC, 5hmC, and 5fC/5caC across hundreds of CpG sites in DNA substrates undergoing oxidation by TET (Fig. 2A). As unmodified cytosine is not a substrate for TET and confound readouts of modification states, we sought a DNA substrate that was highly CpG-methylated. To that end, we leveraged the leaky expression and subsequent self-methylation of a 4.6-kb plasmid encoding the CpG-specific DNA methyltransferase M.MpeI to generate a CpG-methylated substrate containing ∼500 CpG sites in various sequence contexts (Supplementary Fig. S2 and Supplementary File 2.A1–A2). Given our objective to start with a homogenously and highly methylated substrate, we examined global CpG methylation by BS-Seq, finding overall methylation rates of 91 ± 7%. With an added *in vitro* methylation step, global methylation could be further enhanced to 97 ± 2% overall, and the precise starting level of 5mCpG at each site in the substrate could be resolved. The plasmid DNA was sheared to ∼350 bp fragment size, providing the validated substrate, which we term the “context-diverse methylated substrate.” With a substrate candidate established, we focused our analysis on the minimized catalytic domain of TET2, given our interest in interrogating the core active site features dictating sequence preferences. This construct (TET2-CS) is tailored to remove an unstructured insert, yielding a highly active variant that has been the subject of the most extensive *in vitro* biochemical investigations to date [15, 16, 26, 27, 39].

**Figure 2.**
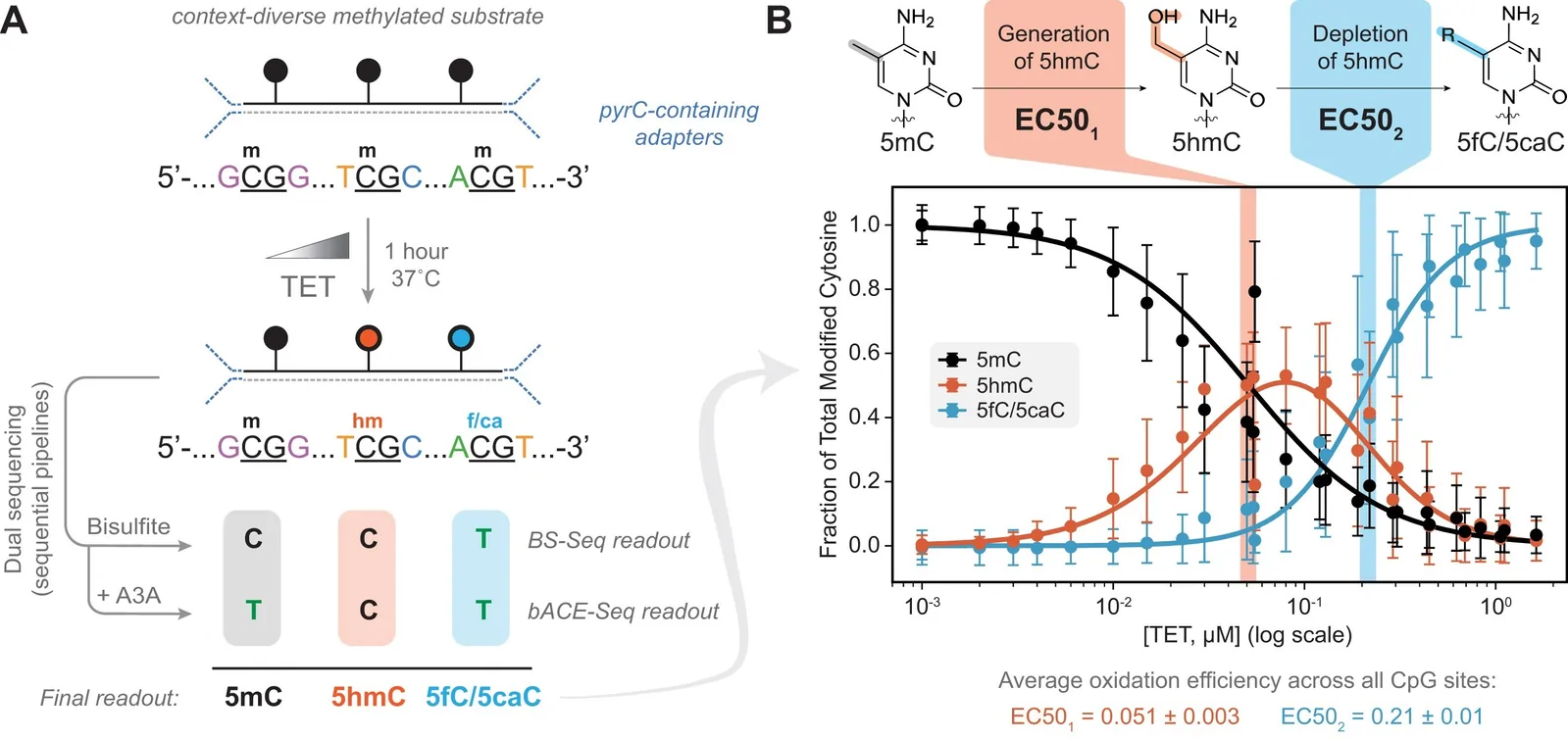
High-throughput assessment of stepwise oxidation of 5mC by TET enzymes. (**A**) Schematic depicting the novel high-throughput, sequencing-based assay used in this study. Assays were conducted with a DNA substrate containing hundreds of methylated CpGs in distinct sequence contexts (the “context-diverse methylated substrate”). The sheared substrate was ligated to modified sequencing adapters (blue dashed lines) containing unnatural cytosines (pyrC, resistant to enzymatic and chemical deamination). After reaction with TET enzyme, a sequential, dual-sequencing pipeline using chemical (bisulfite) and enzymatic (A3A) deamination is used to decode 5mC, 5hmC, and 5fC/5caC at base resolution. (**B**) Semi-log plot of aggregate titration data across all CpGs in substrate. Data were collected from reactions performed in three independent replicate sets, using 80 ng of substrate with TET concentrations ranging from 0 to 1.6 μM. TET concentrations were staggered across replicates to maximize coverage of the dose-response range. Data are plotted as the mean fraction total modified cytosine ± SD. For visualization purposes only, [TET] = 0 μM condition is shown at 10^–3^ μM along the *x*-axis. Curves represent the best fit of the data to a model of stepwise oxidation by TET, with individual points weighted by associated error. The derived parameters EC501 and EC502 are activity constants describing the efficiency of 5mC oxidation (corresponding to the TET concentration at which half of the 5mC has been depleted) and 5hmC oxidation (corresponding to the TET concentration at which half of the 5fC/5caC has been formed), respectively.

To track the reaction progress at CpGs, we employed a sequential workflow generating BS-Seq and bisulfite-assisted APOBEC-coupled epigenetic sequencing (bACE-Seq) libraries constructed from a single TET-oxidized DNA sample (Fig. 2A and Supplementary Fig. S1). BS-Seq and bACE-Seq both rely upon differential deamination of modified cytosine bases, ultimately resulting in selective bases reading out as “T,” while unreacted (non-deaminated) bases read out as “C,” upon sequencing. In BS-Seq, treatment of DNA with bisulfite results in chemical deamination of unmodified cytosine, but not of 5mC or 5hmC. Importantly, 5fC and 5caC are also converted by bisulfite and read as “T.” Thus, with subtraction of the very low (and readily quantifiable) site-resolved levels of unmodified CpGs in the context-diverse methylated substrate, BS-Seq enables the identification of 5mC/5hmC separately from 5fC/5caC. bACE-Seq relies upon the fact that in bisulfite-treated DNA, while 5mC is unreacted, 5hmC is converted to cytosine-5-methylenesulfonate (CMS). Since CMS, but not 5mC, resists enzymatic deamination by the DNA deaminase APOBEC3A (A3A), treatment of bisulfite-converted DNA with A3A allows for subsequent separation of 5mC from 5hmC. Thus, the bACE-Seq library enables identification of 5hmC alone separately from all other cytosines. With quantification of 5fC/5caC via BS-Seq, and quantification of 5hmC via bACE-Seq, bioinformatic subtraction permits calculation of site-specific 5mC levels. Notably, our strategy stands in contrast to prior studies, which have exclusively utilized BS-Seq, either to parse 5mC from 5fC/5caC, without resolving 5hmC, or requiring starting from 5hmC in synthetic DNA substrates (Supplementary Fig. S1) [17, 18].

We reasoned that the ideal dual-sequencing pipeline would allow for the BS-Seq and bACE-Seq samples to be processed from a common sample, splitting at the latest possible step in the pipelines. To potentiate such an approach, the adapters appended to the DNA sample would need to resist both chemical (BS-Seq) and enzymatic deamination (bACE-Seq). In standard BS-Seq, adapters using 5mC in lieu of unmodified cytosine are traditionally used given their resistance to bisulfite; however, as 5mC would react with TET enzymes and unreacted 5mC would be deaminated by A3A, alternatives would be necessary. In recent work, we had demonstrated that unnatural cytosine analogs can potentiate enzymatic approaches, as both 5pyC and pyrC resist enzymatic deamination by A3A [40, 41]. However, whether such unnatural modifications tolerate bisulfite was not established. To consider possibilities, we ligated sheared DNA from the context-diverse methylated substrate to adapters where all cytosines are populated with either C, 5mC, 5pyC, or pyrC, and then attempted library generation before bisulfite, after bisulfite, or after bisulfite and A3A deamination. As anticipated, the control C-containing adapters permit amplification before bisulfite, but not after, given that amplification depends on non-conversion of the adapters. Additionally, as expected, 5mC-containing adapters support library construction after BS-Seq, but not after enzymatic deamination. For our unnatural cytosine-containing adapters, we found that 5pyC-containing adapters are unable to support library generation after bisulfite treatment, presumably due to the reactivity of bisulfite with the 5pyC base. By contrast, pyrC-containing adapters successfully support library generation in both BS-Seq and bACE-Seq, highlighting the resistance of pyrC to both chemical and enzymatic deamination (Supplementary Fig. S4). Armed with a viable approach, we thus integrated pyrC-containing adapters in our split dual-sequencing pipeline, newly allowing us to parse all relevant cytosine states relevant to 5hmC generation and depletion (5mC, 5hmC, and 5fC/5caC) from our substrate.

To study the activity of TET on context-diverse methylated substrate, we reacted the pyrC-adapted DNA substrate at 37°C for 60 min with a range of TET concentrations. We first considered the aggregate data across all CpG sites using the dual BS-Seq and bACE-Seq pipeline (Fig. 2B). For each of the ∼500 starting 5mCpG sites, we calculated the baseline-corrected fractional abundance of 5mC, 5hmC, and 5fC/5caC at each TET concentration, noting that our assay range includes conditions where the majority of 5mCs were converted to 5fC/5caC at the highest concentration of TET examined. We then plotted the mean fractional abundance of each modification state and its variance across all sites. Strikingly, in plotting the fractional abundance of 5mC, 5hmC, and 5fC/5caC at each TET concentration, the two defining oxidation steps and their relationship to the dynamics of 5hmC can now be readily visualized. The TET concentration-dependent changes in 5mC and 5fC/5caC reflect the sigmoidal shapes of monophasic substrate depletion (5mC) and product formation (5fC/5caC), while the net rise and fall of 5hmC is captured by a bell-shaped curve that reflects the balance between opposing changes in 5hmC generation (via oxidation of 5mC) and depletion (via oxidation to 5fC/5caC).

The global oxidation data were fit to a model defined by the three curves representing 5mC consumption, 5hmC generation and decay, and 5fC/5caC generation, from which we obtained overall TET activity constants. To compare the individual steps along the TET-mediated oxidation pathway, we focused our analysis on two fitted parameters that separately capture the efficiency of 5hmC generation (EC50_1_) and depletion (EC50_2_). We defined EC50_1_ as the TET concentration at which half of the 5mC is depleted and EC50_2_ as the TET concentration at which half of the 5fC/5caC is generated. These terms, respectively, reflect the generation of 5hmC and its decay to 5fC/5caC, with lower values corresponding to more efficient TET activity at either step. Using this approach to assess global TET oxidation efficiencies, we obtained an overall EC50_1_ = 0.051 ± 0.003 µM and an EC50_2_ = 0.21 ± 0.01 µM (Fig. 2B and Supplementary File 2.B1). Integrating across 5mCpG sites, we thus observe an average four-fold difference in the requirement for TET enzyme to oxidize 5hmC relative the 5mC. This reactivity gap is within the range of previously reported values of overall relative reactivity determined from simple substrates harboring single or limited reactive CpG sites, providing added validation for this approach to substrate analysis [16, 39].

### 5hmC dynamics are variable across CpG sites

A notable feature of viewing aggregate data from all 5mCpGs across the substrate is the variability in distributions of 5mC, 5hmC, and 5fC/5caC among CpG sites, as captured by the errors of their individual means. A clear strength of our approach is that the BS-Seq and bACE-Seq dual pipeline preserves sequence context information, allowing for the 5hmC dynamics dictated by these coupled oxidation reactions to be simultaneously determined for, and compared between, individual CpG sites in the complex DNA substrate. We thus sought to further explore differences in 5hmC dynamics between individual CpG sites. As expected, when we compared the reaction progress at individual CpG sites, we observed considerable differences in 5hmC generation and depletion (Fig. 3A and B; Supplementary File 2.B2). EC50_1_ values ranged from 9 ± 2 nM among CpG sites where 5hmC was most readily generated (those in the bottom 5% for EC50_1_) to 130 ± 30 nM among CpG sites where 5hmC was least readily generated (those in the top 5% for EC50_1_), suggesting a >10-fold difference in the enzyme requirements for 5mC consumption between the most reactive and least reactive sites. Similarly, EC50_2_ values ranged from 50 ± 10 nM among CpG sites where 5hmC was most readily depleted (those in the bottom 5% for EC50_2_) to 0.9 ± 0.3 µM among CpG sites where 5hmC was least readily depleted (those in the top 5% for EC50_2_), highlighting a large range of differences with regard to 5hmC depletion relative to 5hmC generation (Supplementary Fig. S5).

**Figure 3.**
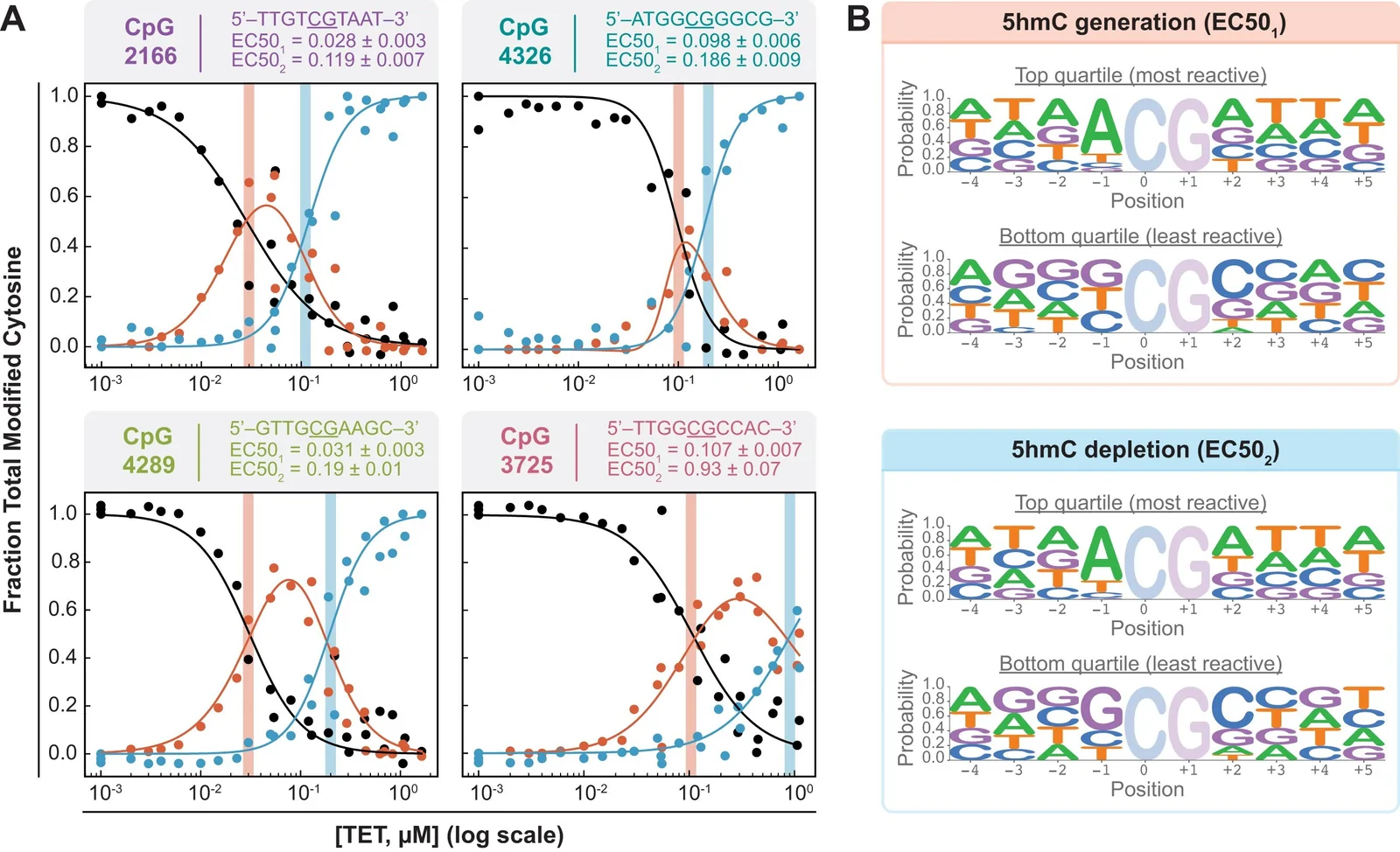
Sequence context alters reactivity of 5mC and 5hmC. (**A**) Representative titration curves of four individual CpG sites in the context-diverse methylated substrate showing distinct dynamics of stepwise oxidation by TET. As in Fig. 1C, for visualization purposes only, the [TET] = 0 μM conditions are shown at 10^–3^ μM along the *x*-axis. The flanking sequence context around selected sites is shown with the relevant CpG underlined. (**B**) DNA probability logos demonstrating the base representation of CpG 10-mers among sites of top quartile and bottom quartile for both 5hmC generation and depletion.

As neither the position of the CpG within the substrate nor the density of neighboring CpGs demonstrated strong correlations with EC50_1_ or EC50_2_ (Supplementary Fig. S6 and Supplementary File 2.C1–C2), we focused attention on the identity of the bases flanking each CpG as the variable most likely to explain the observed differences in CpG-specific 5hmC generation and depletion efficiencies. We defined the target 5mCpG sites as the 0 and +1 positions and cataloged bases on either side of these target sites, including upstream 5′-bases (positions −4 to −1) and downstream 3′-bases (positions +2 to +5) relative to the target. For both EC50_1_ and EC50_2_, an initial analysis of the frequency of A, C, G, and T at the more distal positions (−4 to −2 and +3 to +5 relative to the central CpG) showed small differences in the distribution of bases between the top and bottom quartile (Fig. 3B), aside from a general trend toward overrepresentation of G in the 5′-region and C in the 3′-region relative to the target CpG. By contrast, the positions immediately flanking the CpG, positions −1 (${\mathrm{\mathbf{N}}\underline{C}G}$) and +2 (${\rm \underline{C}G{\mathbf{N}}}$), accounted for the majority of the effects of sequence context on the variability of 5hmC dynamics. Of note, when considering the “4-mer” (${\rm {\mathbf{N}}\underline{C} G {\mathbf{N}}}$) sequences, these were well represented across our substrate (range 18–43 per 4-mer) and showed no significant differences in starting methylation levels (Supplementary Fig. S2), offering sufficient observations for more detailed investigation. We thus decided to focus further detailed analysis on the immediate upstream and downstream bases flanking the CpG for understanding the sequence context preferences of TET for 5hmC flux.

### Flux through 5hmC depends on sequence context

To determine the influence of sequence context on the efficiency of 5hmC generation and depletion, we first stratified the data for EC50_1_ and EC50_2_ according to the base identity at either the −1 position or at the +2 position for each CpG site (Fig. 4A and B). Consistent with prior reports of TET preference for oxidizing ${\mathrm{\mathbf{A}}\underline{C}G}$ sites [17, 18], we found that 5hmC was generated ∼3-fold more efficiently at CpGs flanked by an A at the −1 position than at other CpG sites (${\mathrm{\mathbf{B}}\underline{C}G}$, B = C, G, or T). Similarly, we observed that 5hmC depletion was ∼2–4-fold more efficient at ${\mathrm{\mathbf{A}}\underline{C}G}$ sites than at ${\mathrm{\mathbf{B}}\underline{C}G}$ sites, highlighting that preferred substrates for 5hmC generation and depletion are similar. By contrast, while the efficiency of 5hmC generation was similar across ${\mathrm{\mathbf{C}}\underline{C}G}$, ${\mathrm{\mathbf{G}}\underline{C}G}$, and ${\mathrm{\mathbf{T}}\underline{C}G}$ sites, the efficiency of 5hmC depletion was more variable among these same three sequence contexts with ${\mathrm{\mathbf{G}}\underline{C}G}$ resisting oxidation the most (${\mathrm{\mathbf{T}}\underline{C}G}$ > ${\mathrm{\mathbf{C}}\underline{C}G}$ > ${\mathrm{\mathbf{G}}\underline{C}G}$). Thus, using a dual-sequencing pipeline to resolve 5hmC from 5fC/5caC highlights differences in the sequence context preferences of 5mCpG oxidation relative to 5hmCpG oxidation that were masked by prior studies’ focus on 5fC/5caC endpoints. Downstream of the CpG, the effects of base identity at the +2 position were less prominent. For both the generation and depletion of 5hmC, efficiencies were comparable within error across all four sequence contexts (CG**A**, CG**C**, CG**G**, and CG**T**), with a very modest bias against CG**C** sites.

**Figure 4.**
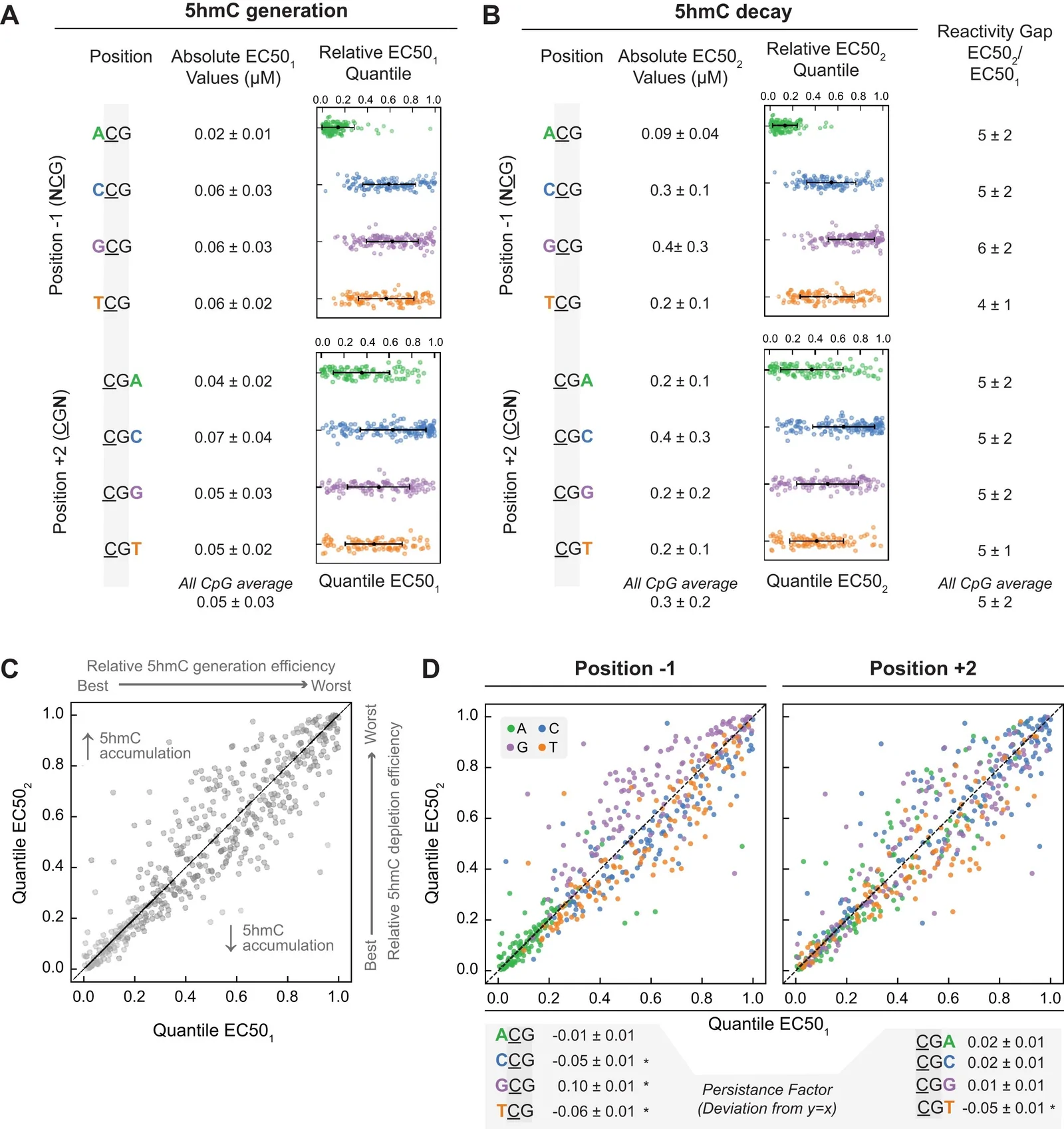
The efficiencies of 5hmC generation and depletion depend on the bases flanking the CpG site. (**A, B**) Shown are the absolute EC501 or EC502 means ± SD, along with the ratio of EC502/ EC501 (at far right) grouped by base identity at the −1 and +2 positions. For relative quantile plots, individual points represent the quantile rank of the EC501 or EC502 value. Group means ± SD for CpG sites grouped by base identity at the −1 and +2 positions are shown overlaid in black. (**C**) Comparison scatter plots in which each CpG site is represented by a point with coordinates corresponding to its rank orders among all CpGs for EC501 and EC502. For points above the line, at that position 5hmC is less reactive than 5mC when compared to other sites. (**D**) The same plots as in panel C, now color-coded according to the base identity at position −1 (left) or position +2 (right). The persistence factor reports on whether the cluster of points for a given variable fall above or below the line of identity, with * denoting *P* < .0005 for deviation of the intercept from 0 for the best-fit line with slope = 1.

A key feature of our high-throughput assay is the ability to assess the relative efficiencies of 5hmC generation and depletion at each CpG. Leveraging this wealth of correlated data, we moved from examination of the absolute individual values to a rank-ordered analysis. Each of the ∼500 CpGs was ranked by quantile according to its EC50_1_ and EC50_2_ values, with the rank order values (range 0.0–1.0) largely reflecting the trends observed with absolute values with regards to preferences for the −1 and +2 positions (Fig. 4A and B). A strength of examining the data through a relative lens is that it facilitates comparison of 5hmC generation efficiencies to 5hmC depletion efficiencies, reflecting measurements of flux in the procession through 5hmC at each CpG site. To this end, we plotted the EC50_1_ quantile and EC50_2_ quantile of individual CpGs against one another (Fig. 4C and D). In this plot, the line of identity represents the positioning of CpGs that show similar ranking, relative to other CpGs, for 5hmC generation as for 5hmC depletion. Sites at which TET tends to stall at 5hmC fall above the line of identity, as 5hmC generation is disproportionately more efficient than 5hmC depletion at these sites relative to other CpGs (thus leading to relative 5hmC accumulation, as noted in Fig. 4C). By contrast, CpGs positioned below the line of identity represent sites at which, relative to other CpGs, TET more readily processes past 5hmC to 5fC/5caC (resulting in relative 5hmC depletion, as noted in Fig. 4C). Thus, the positioning relative to the line of identity corresponds to the size of the reactivity gap between 5mCpG and 5hmCpG at a given site.

Overall, we observed that the quantiles for EC50_1_ and EC50_2_ strongly correlated with each other (best-fit slope = 0.90 ± 0.02, Spearman ρ = 0.90, *P* < .0005). To incorporate the sequence context variable into this analysis, each CpG was delineated according to the identity of the base at the −1 or +2 position (Fig. 4D). Visualizing the data in this manner, when color-coded by base identity at the −1 position (Fig. 4D, left), several clusters of data became readily apparent. One group of points, clustered tightly around the line of identity at the bottom left of the plot, represents the most reactive substrates and largely encompasses CpGs flanked by A at the −1 position (${\mathrm{\mathbf{A}}\underline{C}G}$, green dots). With few exceptions, for CpGs in this context, 5hmCs generated and depleted were both of high and similar efficiency relative to other sites, as demonstrated by the fact that the intercept for the best-fit line with slope constrained at 1 for this subset of CpG sites is approximately zero and thus does not significantly deviate up or down from the line of identity. We refer to this deviation as the “5hmC persistence factor” or PF, the magnitude of which corresponds to the degree to which 5hmC is more persistent (for PF > 0) or less persistent (for PF < 0) than typical (Supplementary Fig. S7). A second notable cluster was dominated by CpGs flanked by G at the −1 position (purple dots). This cluster spanned a wider range of EC50_1_ values but fell almost exclusively above the line of identity (PF = 0.10 ± 0.01, *P* < .0005). Therefore, regardless of the efficiency with which 5hmC was generated at these CpG sites, subsequent depletion of 5hmC was disproportionately less efficient relative to other CpGs, a reactivity profile associated with a significant relative accumulation of 5hmC at ${\mathrm{\mathbf{G}}\underline{C}G}$ sites. Quantitatively, this difference corresponded to a six-fold lower absolute reactivity for ${\mathrm{\mathbf{G}}\underline{5hmC}G}$ than for ${\mathrm{\mathbf{G}}\underline{5mC}G}$, a widened gap relative to CpGs within other sequence contexts (Fig. 4B). Interestingly, at CpGs flanked by T at the −1 position, depletion of 5hmC was in many cases disproportionately more efficient, corresponding to a significant deviation from the line of identity of PF = −0.06 ± 0.01 (*P* < .0005) and a four-fold difference in absolute reactivity with ${\mathrm{\mathbf{T}}\underline{5mC}G}$ than with ${\mathrm{\mathbf{T}}\underline{5hmC}G}$. Similar behavior was observed at CpGs flanked by C at the −1 position (PF = −0.05 ± 0.01, *P* < .0005). Of note, given that EC50_2_ is always greater than EC50_1_, we considered the possibility that alternative ways of examining the positional data might be useful (Supplementary Fig. S7). To this end, we also compared EC50_1_ against either the Δ(EC50_2_–EC50_1_), which represents the additional TET required to convert half of 5hmC relative to that needed for 5mC oxidation, or the ratio of EC50_2_/EC50_1_. In both alternative analyses, the clustering patterns based on the −1 position remained evident, supporting the utility of our initial approach.

When a similar analysis was performed to look at the effects of the +2 position on the accumulation or depletion of 5hmC (Fig. 4D, right), we found that while all groups had comparable absolute reactivity gaps of ∼5 (Fig. 4B), turnover of 5hmC was relatively favored at CG**T** sites based on a significant deviation from the line of identity of PF = −0.05 ± 0.01 (*P* < .0005). Although the effect size is smaller than that observed with −1 base identity, we suspected that the observed effects of the +2 position might be partially tempered by influence from the −1 base, prompting us to next closely examine the interactions between these two positions.

### Base flipping of CpGs may influence TET substrate preferences

In addition to highlighting differential effects of sequence context on 5mC and 5hmC oxidation, our analysis focused our attention on the −1 and +2 positions when considering each position independently. Given potential mechanisms by which these two positions may interact with the central CpG, we aimed to examine their combinatorial effects by stratifying the data by the “4-mer” sequence context of each CpG (Fig. 5A and B). Visualizing the data in this manner, in contrast to the merged data, the effects of the +2 position can be more clearly ascertained. For both EC50_1_ and EC50_2_, the −1 position effects remain apparent, with ${\mathrm{\mathbf{A}}\underline{C}GN}$ being consistently favored relative to ${\mathrm{\mathbf{B}}C\underline{G}N}$ (B = C, G, or T). Within each series, however, we also observe a significant and consistent preference at the +2 position with a downstream A preferred and a downstream C disfavored (Supplementary Table S1), which aligns with our hypothesis that the considerable -1 position preferences were masking cooperative effects from the +2 position. At a quantitative level, when separating based on −1 position, the presence of a +2 A results in a ∼1.3–1.5-fold lower EC50_1_ and EC50_2_, while the presence of a +2 C results in a ∼1.5–2-fold higher EC50_1_ and EC50_2_, suggesting an overall similar impact on 5hmC generation and 5hmC depletion.

**Figure 5.**
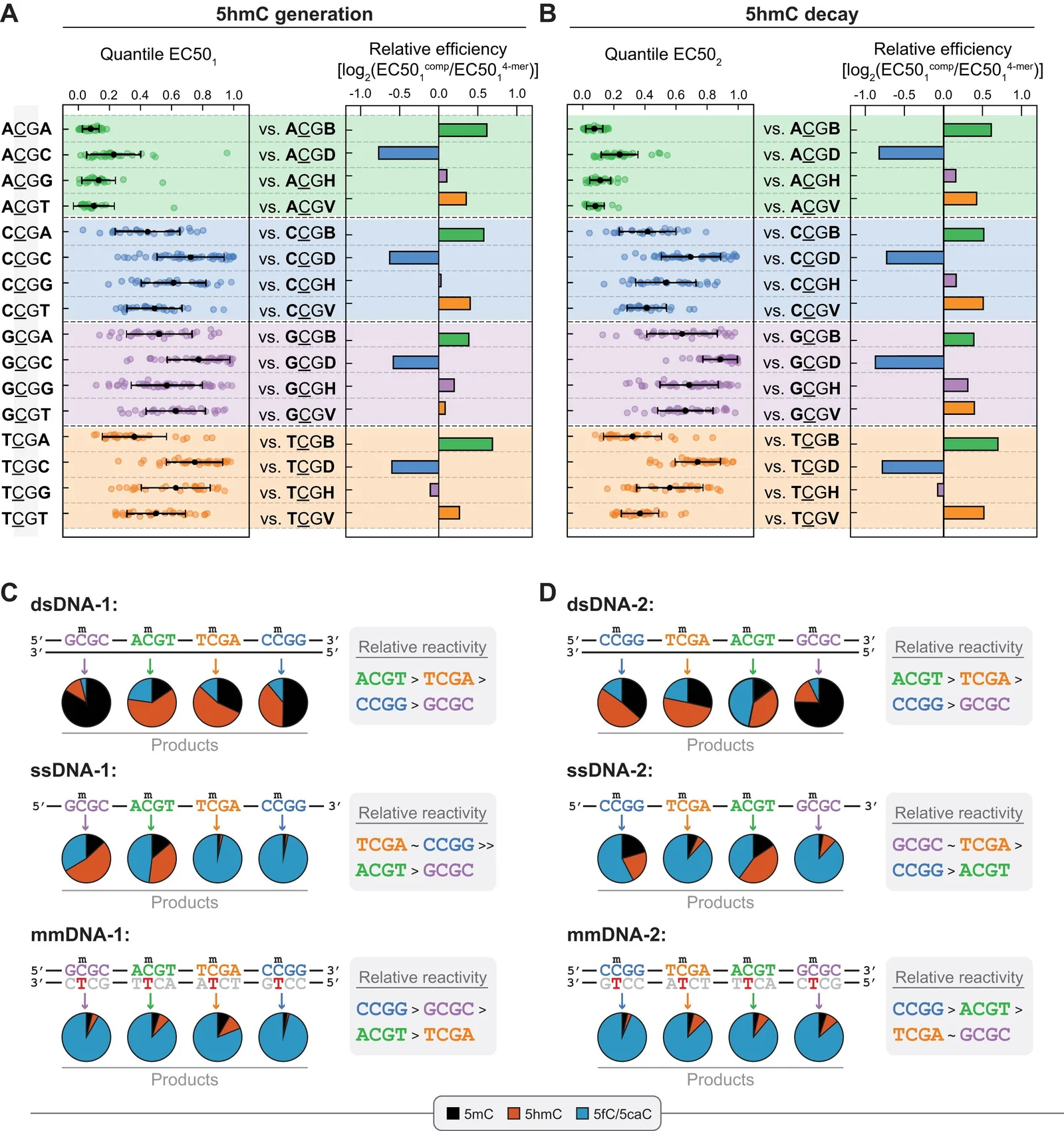
Tetranucleotide sequencing context influences TET activity, with predictable effects confined to duplex DNA. (**A, B**) Combinatorial effects of base identity at the −1 and +2 positions on the relative efficiency of 5hmC generation and depletion, with data grouped according to the sequence context of the central CpG 4-mer. At left, individual data points are plotted, with group means ± SD are overlayed in black. At right is the relative efficiency as measured by the relationship to a comparison group (B = C/G/T, D = A/G/T, H = A/C/T, V = A/C/G) (**C, D**) Model oligonucleotide substrates were designed containing four 5mCpG sites in distinct sequence contexts. Oligonucleotides, in either duplex (dsDNA-1, dsDNA-2), single-stranded (ssDNA-1, ssDNA-2), or mismatch form (mmDNA-1, mmDNA-2) were reacted with a fixed TET concentration (0.5 μM, 1 h). Shown are pie charts denoting the average resulting 5mC, 5hmC, and 5fC/5caC at each site derived from at least three independent replicates and quantified using the dual-sequencing pipeline. Shown at right are the relative reactivities of the four sites, where dsDNA substrates follow predictable trends while ssDNA and mmDNA substrates do not.

The analysis of −1 and + 2 position preferences highlights cooperativity between these two positions in shaping oxidation of the interior 5mCpG. The preference for A at the −1 position and A or T at the +2 position led us to consider whether weaker flanking A:T base pairs could alter the kinetics of base flipping required for 5mC to access the TET active site. The energetics of base flipping involves overcoming both the stabilizing effects of interstrand hydrogen bonding and intrastrand base stacking interactions [42–50]. If sequence context preferences are partly driven by the ease of base flipping, then weakening base-pairing constraints should increase activity while diminishing sequence-dependent differences, particularly at CpGs within contexts that are poor substrates in fully complementary dsDNA. To test this specific prediction of a base-flipping model, we designed a synthetic ssDNA oligonucleotide harboring four methylated cytosines within palindromic 4-mer contexts (ACGT, CCGG, GCGC, TCGA), which we selected because they spanned the full range of TET activities observed with the context-diverse methylated substrate. We partnered this ssDNA with an unmethylated complement to yield hemi-methylated, fully complementary dsDNA. This substrate, named dsDNA-1, was then reacted with a fixed concentration (0.5 µM) of TET2-CS, and the dual-sequencing pipeline was used to quantify 5mC, 5hmC, and 5fC/5caC (Fig. 5C and Supplementary Fig. S8). With dsDNA-1, the relative reactivity of each CpG in the dsDNA substrates matched the expected reactivity order based on our analysis of the context-diverse methylated substrate (ACGT > TCGA > CCGG > GCGC). Strikingly, we observed an overall increase of TET activity with ssDNA-1 and a significant change in the reactivities of individual CpGs in this substrate, now more modestly favoring TCGA ∼ CCGG >> ACGT > GCGC. To ensure that the changes in reactivities were not influenced by the specific positioning of the 4-mers within the sequence, we designed a second set of oligonucleotides (dsDNA-2, ssDNA-2) in which the order of the 4-mers was reversed (Fig. 5D). With the dsDNA-2 substrate, we again found that the reactivity in the duplexed substrate was predictable based upon our analysis of the context-diverse methylated substrate. With the ssDNA-2 substrate, however, the order of reactivity of individual CpGs, while differing somewhat from that of ssDNA-1, was similarly perturbed relative to the consistent and well-defined preferences seen with dsDNA.

The above comparison of the matched dsDNA and ssDNA substrates suggested that the loss of interstrand hydrogen bonding and diminished base stacking interactions increased overall reactivity and altered its sequence context dependence. To further explore the mechanisms at play, we designed one further permutation of the substrate series, using a complementary strand with targeted 5mC:T mismatches at each of the four CpG sites in the oligonucleotide. We validated that the mismatched complement strand did not impact substrate duplexing (Supplementary Fig. S9) and compared reactivity to that of the matched dsDNA and ssDNA substrates. We found that the overall reactivity of the mismatched DNA (mmDNA-1, mmDNA-2) was very high and closer to that of the ssDNA than the dsDNA, suggesting that the low barrier to eversion of the 5mC from the duplex DNA accounted for a significant part of the overall reactivity. As with the ssDNA substrates, the reactivity preferences, which were predictable and ordered with dsDNA substrates, were again no longer predictable with mmDNA. Taken together, these observations highlight the dependence of TET sequence context preferences on the local base-pairing environment that contributes to the energetic landscape of base flipping.

### Generalizability of sequence context preferences using orthogonal substrates and assays

Our analysis suggested that sequence preferences were generalizable between the context-diverse methylated substrate and the designed oligonucleotide dsDNA substrates. Seeking to confirm whether the observed preferences would be reproducible with an alternative and biologically representative DNA substrate, we aimed to also examine CpG sites based on the sequence of the murine KvDMR locus, a mammalian TET-dependent ICR [51]. The ICR sequence was inserted into the self-methylating M.MpeI-encoding plasmid to generate a new larger plasmid (7119 bp) harboring >200 additional 5mCpG sites within the KvDMR insert. The new substrate, which we term the “methylated KvDMR-containing substrate,” was validated to again be >97% CpG-methylated on average (Supplementary File 2.A3–A4). With this alternative substrate, we evaluated using our high-throughput TET titration assay to probe the sequence-context specific patterns of 5hmC generation and depletion at CpG sites within the KvDMR locus (Supplementary File 2.B3–B4, Supplementary Table S2). Analyzing across the substrate, we observed that the aggregate TET activity across all CpGs was slightly blunted (EC50_1_ = 0.18 ± 0.01 μM, EC50_2_ = 0.43 ± 0.02 μM) relative to that observed for the original context-diverse methylated substrate (Supplementary Fig. S10A). Nonetheless, the specific reactivity trends across CpGs in different sequence contexts were highly reproducible, suggesting similar *in vitro* behavior of TET acting on substrates within mammalian- and bacterial-derived DNA sequences. With the CpGs in the KvDMR locus, we again observed a clear preference for **A**CG sites for both 5hmC generation and 5hmC depletion (Supplementary Fig. S10B). Likewise, 5hmC depletion and turnover propensities were in excellent agreement with those observed for the original context-diverse methylated substrate, with 5hmC at **G**CG sites showing a lower relative reactivity compared to **T**CG and **C**CG sites (Supplementary Fig. S10C).

Notably, in this study, we favored analysis with enzyme titration, given that time-dependent self-inactivation of TET enzymes is known to impact kinetic analysis [3]. While recognizing this constraint, to further validate our findings using a kinetic approach, we also assessed sequence context dependence as a function of time to examine whether enzyme-concentration dependent trends remain similar (Supplementary File 2.D1–D2). To do so, we quantified the fraction of 5mC, 5hmC, and 5fC/5caC at each CpG site from initiation of the TET reaction to time points as early as 0.5 min and out to ∼1 h. We plotted the mean and distribution of modifications at each CpG site and observed an expected pattern with initial decay of 5mC, accumulation of 5hmC peaking at ∼3 min, and ongoing accumulation of 5fC/5caC out to late time points (Supplementary Fig. S11A). To examine correspondence between time-resolved and concentration-dependent datasets, we plotted each CpG as a point with coordinates corresponding to the depletion of 5mC at that site along the *y*-axis and the EC50_1_ quantile of that same site, as determined from the TET concentration-dependent titration assays, along the *x*-axis (Supplementary Fig. S11B). We similarly plotted the accumulation of 5fC/5caC at each time point against the EC50_2_ quantile for each site. With both plots, we observed a strong correlation, wherein CpG sites identified as more favorable in enzyme titration assays displaying faster oxidation kinetics, validating that our enzyme concentration-dependent approach to data analysis also reflects kinetic differences. Furthermore, when the data were stratified by base identity at the −1 or +2 position (Supplementary Fig. S11C), the kinetic differences between sequence contexts again mirrored the sequence context preferences emerging from the enzyme titration assays. These findings demonstrate the robustness of EC50_1_ and EC50_2_ as useful proxies for comparative analyses of TET kinetics, with the enzyme titration assays also allowing for simpler modeling and elimination of time-dependent loss of TET activity as a potential confounding factor.

### Genomic hydroxymethylome landscapes show sequence context bias

With our approach having revealed *in vitro* sequence context preferences for TET, we next considered whether there is evidence of sequence context bias in hydroxymethylome landscapes from mammalian genomes. While the expectation is that *in vivo* 5hmC patterns will be the product of complex factors in cells, we considered the possibility that intrinsic, *in vitro* preferences of TET enzymes could contribute to some aspects of 5hmC patterning. To explore this possibility, we analyzed publicly available datasets obtained from the GEO, focusing on two cell types in which 5hmC has been well characterized: mouse excitatory neuronal cells and ESCs. Our analysis included BS-Seq datasets, which profile 5modC (5mC/5hmC), and either TAB-Seq or ACE-Seq datasets, both of which profile 5hmC alone (Supplementary File 2.E1–E4).

We first analyzed 5modC in the neuronal and ESC datasets to ask whether modified CpGs show bias as a function of the neighboring sequence context. Focusing on the −1 or +2 bases, we plotted the frequency with which 5modC is found relative to the genome-wide prevalence of the same ${\mathrm{\mathbf{N}}\underline{C}G}$ or ${\rm\underline{C}G{\mathbf{N}}}$ sequence motif. We found that, across sequencing modalities and in both cell types, 5modC bases show no evidence of sequence bias as a function of either the −1 or +2 position (Fig. 6). Rather, 5mC and 5hmC taken together are found within flanking bases at levels that are proportional to the genomic prevalence of CpGs. As the majority of the 5modC is 5mC in both neurons and ESCs, these results suggest the absence of bias in the prevalence of the 5mC substrate for TET-mediated oxidation.

**Figure 6.**
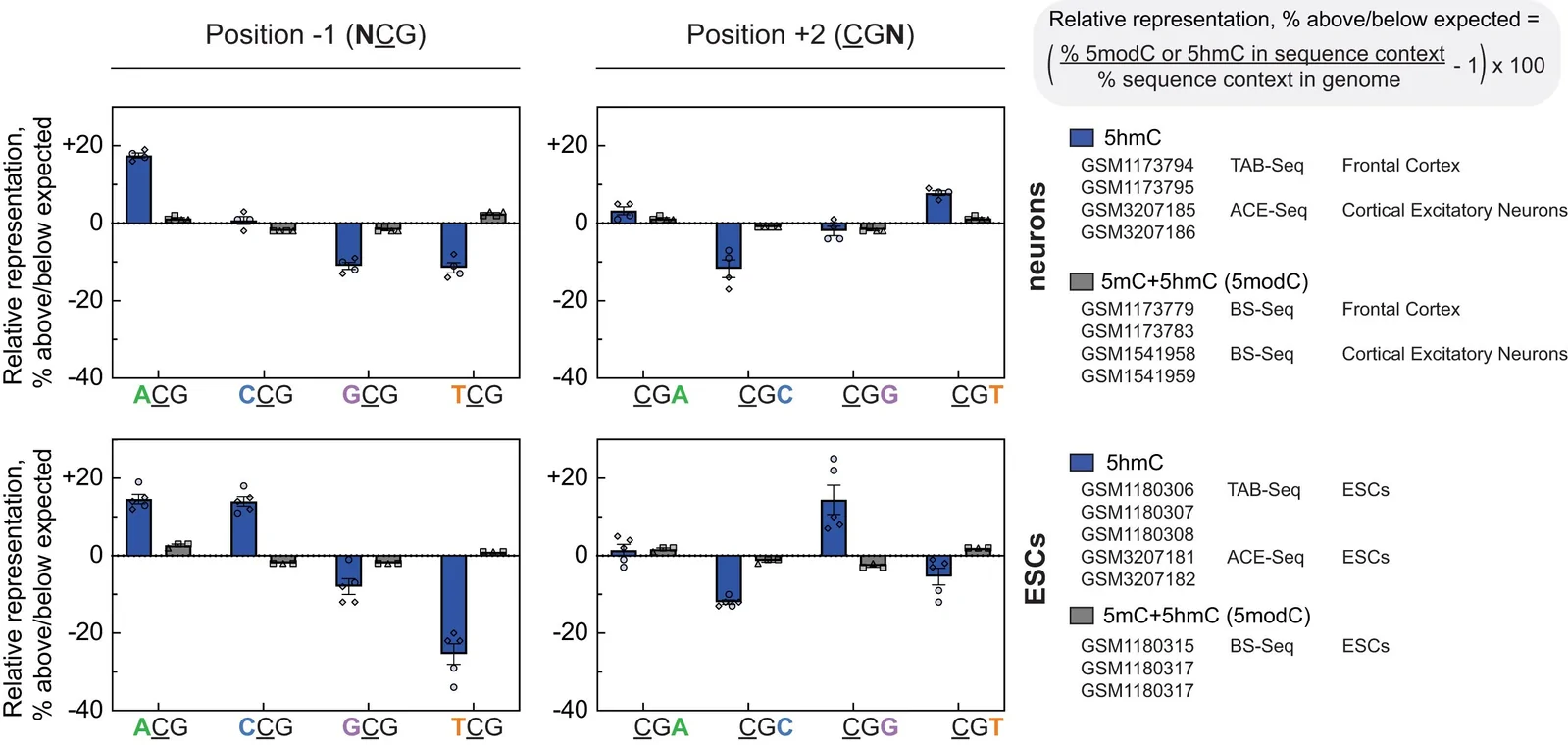
Sequence context dependence of 5mC and 5hmC in genomic DNA. Base-resolution profiles of 5modC (5mC/5hmC) or 5hmC alone in genomic DNA (gDNA) from mouse excitatory neurons or mouse ESCs were reanalyzed incorporating sequence context information. Genomic CpG sites at which modified bases were present were grouped according to the base identity at the −1 or +2 position. Plotted is the enrichment or depletion of 5modC or 5hmC relative to the frequency of the sequence context in the genome overall. Bars represent averages of individual datasets with ± SD noted. Shapes represent replicates of different datasets and sequencing pipelines. Square, triangle = BS-Seq; diamond = TAB-Seq; circle = ACE-Seq.

By contrast with 5modC, we readily observed biases in the prevalence of 5hmC located within different flanking sequence contexts (Fig. 6). Specifically, we observed an effect of the −1 position wherein, in neurons, 5hmC is overrepresented at ${\mathrm{\mathbf{A}}\underline{C}G}$ sites and underrepresented at ${\mathrm{\mathbf{G}}\underline{C}G}$ and ${\mathrm{\mathbf{T}}\underline{C}G}$ sites. The pattern was similar in ESCs, except that 5hmC was additionally overrepresented at ${\mathrm{\mathbf{C}}\underline{C}G}$ sites. An effect of the +2 position was also noted, wherein 5hmC was overrepresented at ${\rm\underline{C}G{\mathbf{T}}}$ (neurons) or ${\rm\underline{C}G{\mathbf{G}}}$ sites (ESCs) and underrepresented at ${\rm\underline{C}G{\mathbf{C}}}$ sites. Notably, as our selected datasets for mapping 5hmC include orthogonal methods (TAB-Seq and ACE-Seq), the observed bias in 5hmC prevalence is likely not due to any intrinsic bias in the methods used. Therefore, in contrast to 5modC bases, 5hmC localization shows evidence of biases relative to genomic distribution, with enrichment of 5hmC in specific sequence motifs, including a consistent enrichment at ${\mathrm{\mathbf{A}}\underline{C}G}$ sites that aligns with some of the observed *in vitro* preferences for 5mC oxidation.

Given the evidence that 5hmC prevalence, unlike that of 5mC, can vary as a function of sequence context in neurons and ESCs, we also explored whether these patterns specifically manifest in TF binding sites. Using ACE-Seq data from neurons and ESCs, we identified all TF binding sites known to potentially contain a CpG and overlaid the level of 5hmC at the associated site on a Principal Component Analysis (PCA) plot that captures and parses the diversity of TF binding site sequences (Supplementary Fig. S12). We observed that the 5hmC levels in TF binding sites appeared to track with those observed globally, with TF binding sites harboring ${\mathrm{\mathbf{A}}\underline{C}G}$ or ${\rm\underline{C}{\mathbf{G}}T}$ containing high 5hmC levels in both neurons and ESCs, with added enrichment of 5hmC in ${\mathrm{\mathbf{C}}\underline{C}G}$- or ${\rm\underline{C}G{\mathbf{G}}}$-containing TF binding sites in ESCs. Thus, the differences in 5hmC distribution as a function of sequence context can also manifest in specific genomic elements, with functional implications in controlling gene regulatory networks.

## Discussion

Prior biochemical studies on the intrinsic substrate preferences of TET *in vitro* have offered a view into the enzymatic properties that contribute to gene regulation in the more complex *in vivo* setting [17, 18, 28, 39, 52]. Nonetheless, limitations in the ability to parse the different oxidation products resulting from 5mC have constrained our ability to understand factors that specifically govern 5hmC accumulation versus its decay. Discrimination between these steps is of particular importance given the central role of 5hmC as an independent epigenetic mark and a branch point between replication-dependent and replication-independent DNA demethylation pathways. Toward this objective, we leveraged a high-throughput, dual-pipeline sequencing-based assay that permits simultaneous examination of hundreds of individual CpG sites within a DNA substrate. The approach makes use of differentiation between modified cytosine bases using chemical (bisulfite) and enzymatic (A3A) deamination, together with dual-resistant pyrC-containing adapters, to parse oxidation of 5mC to 5hmC from oxidation of 5hmC to 5fC/5caC. Using this approach, consistent with past studies, we find that ${\mathrm{\mathbf{A}}\underline{C}G}$ is preferred as a substrate for both 5mC and 5hmC oxidation [17, 18], pointing to the robustness of the finding with different substrates and assay approaches. By parsing 5hmC generation from 5hmC depletion, here we also newly describe the dependence of 5hmC turnover on sequence context. We found that ${\mathrm{\mathbf{G}}\underline{C}G}$ sites are characterized by a lower relative reactivity of 5hmC, while ${\mathrm{\mathbf{T}}\underline{C}G}$ and ${\mathrm{\mathbf{C}}\underline{C}G}$ sites show higher relative reactivity. We further reveal synergy between the −1 and +2 positions in sequence context dependence, with the −1 position preferences masking an otherwise evident favored oxidation of CpGs flanked by A at the +2 position or disfavored oxidation of CpGs flanked by C at the +2 position. We also offer support for the conjecture that these sequence preferences could result in part from duplex base pairing and the requirement for base flipping in TET-mediated oxidation, given that the consistent trends are predictable in model dsDNA substrates but lost in ssDNA substrates. Finally, by reexamining established 5hmC maps from neurons and ESCs, we find that 5hmC shows bias in its prevalence as a function of sequence context, with 5hmC consistently accumulating in ${\mathrm{\mathbf{A}}\underline{C}G}$ above anticipated levels, while 5modC shows no such bias.

### Observed sequence preferences are compatible with a base-flipping mechanism

The finding of sequence preferences in TET-mediated oxidation of 5mC could be explained by multiple mechanisms, including discrete molecular contacts between enzyme and substrate or within the substrate, or other biophysical properties such as breathing of nucleobases within DNA. Regarding discrete molecular contacts, a recent structural model of TET2 in complex with DNA harboring 5mC suggested that, when the target 5mC base is flanked by an A at the −1 position, the orphaned opposite-strand G can adopt an anti-orientation and donate a hydrogen bond to its neighboring +2 T, a stabilizing interaction that is not formed when the base undergoing extrahelical flip is flanked by C or G at the −1 position [18]. Similarly, the model posited that a specific interaction with R1302 of TET2-CS could account for 3′-specificity through direct interactions.

Alongside these explanations, our data suggest that base flipping could provide an added useful biophysical lens for considering differences between substrates. Base flipping is a feature common to many DNA modifying enzymes, including DNA MTases and TET enzymes, whereby the base being modified is everted out of the DNA duplex and into the enzyme active site [53]. In the case of TET enzymes, multiple interactions present in duplex DNA are lost as part of the catalytic cycle. These disrupted interactions include hydrogen bonding with the opposite-strand CpG dyad and stacking interactions with the nucleobases neighboring the extrahelical base. Sequence-specific differences in the energetic cost to base flipping have been reported for other DNA-modifying proteins that utilize base flipping [49, 54, 55] and could similarly offer a mechanistic explanation for the sequence context preferences of TET. Our study demonstrates that the observed patterns of 5hmC generation and depletion extended from the context-diverse methylated substrate to double-stranded oligonucleotides but become perturbed when base-pairing at the target CpG is altered and are generally diminished either by the absence of an opposite strand entirely (ssDNA) or by the presence of a 5mC:T mismatch across each target CpG (mmDNA). mmDNA, which disrupts Watson–Crick base-pairing at the target site while minimizing structural perturbations to the duplex, shows particularly high reactivity overall, which could reflect a combination of the low barrier to eversion of the target 5mC and preservation of optimal contacts with flanking dsDNA. These findings suggest that considering molecular contacts between the enzyme and the target strand alone could be complemented by considering biophysical properties of the substrate itself, as the dynamics of accessing cytosine in stable duplex DNA are also contributing factors to substrate discrimination.

### Sequence preferences for 5mC and 5hmC oxidation

The intricacies of multi-step oxidation by TET enzymes raise added questions regarding factors that could lead to different sequence preferences for 5mC versus 5hmC oxidation. Our approach allowed us to study the progression of hundreds of individual 5mC bases to 5hmC and then on to 5fC/5caC, with the results highlighting that sequence preferences for the generation of 5hmC differ from those of 5hmC depletion. Most notably, we find that 5hmC in ${\mathrm{\mathbf{G}}\underline{C}G}$ contexts shows less reactivity than in ${\mathrm{\mathbf{C}}\underline{C}G}$ and ${\mathrm{\mathbf{T}}\underline{C}G}$ contexts, despite the fact 5mC reactivity is similar across these contexts. In considering how reaction products progress from 5mC onwards, it remains unresolved whether DNA or individual CpG sites are released from TET between cycles of oxidation [28, 56]; however, across all models, the TET mechanism requires base-flipping dynamics, as the co-substrate α-KG is buried deep relative to the everted nucleobase, and the resulting succinate product must be exchanged for new α-KG between steps. In considering mechanisms that could explain our findings, we posit that differential impacts on 5mC versus 5hmC could be attributable to changes in base flipping, either due to altered interactions with the opposite-strand CpG or to base-stacking impacts from flanking bases. Regarding the opposite-strand CpG interactions, DNA methylation is well-known to increase the melting temperature of duplex DNA. 5hmC reverses this effect through relatively weaker interactions with the opposite-strand CpG, offering one mechanism that explains differences in the accessibility of 5mC versus 5hmC in different sequence contexts. Regarding flanking base effects, given that 5hmC turnover was more efficient when the central CpG was flanked by a C or T at the −1 position relative to G at this position, it is reasonable to speculate that the weaker pyrimidine base stacking interactions [57] may differentially impact 5hmC base flipping. In support of this possibility, a prior NMR study examined base pair opening dynamics in the Dickerson–Drew dodecamer ([CGCGAATTCGCG]_2_) harboring unmodified or modified cytosine (noted by C). In this study, the rate of opening of adjacent base pair varied depending on the modification state of the target cytosine, with the presence of 5hmC increasing the base-flipping rate by three-fold relative to unmodified C [58]. While this type of comprehensive analysis has not been performed for CpGs across all sequence contexts, the results highlight a role for the flanking DNA sequence in differentially impacting base flipping of modified cytosines, which could help rationalize our observations.

### 5hmC prevalence shows sequence bias *in vivo*

In our *in vitro* work, focusing on the enzyme-substrate interactions enables a description of the sequence context preferences intrinsic to TET enzymes alone. Within cells, a much larger set of influences are anticipated to shape the landscape of DNA modifications, thus adding complexity. Prompted by our more detailed understanding of *in vitro* TET substrate preferences, here we also newly analyzed prior base-resolution epigenetic sequencing studies on mammalian genomes. Examining two different cell types, neurons and ESCs, we found that stable 5hmC levels, but not 5modC levels, are biased as a function of sequence context. Although aspects of these patterns differed by cell type, 5hmC was consistently more prevalent in ${\mathrm{\mathbf{A}}\underline{C}G}$ contexts, consistent with *in vitro* preferences for 5mC oxidation. Interestingly, these genomic datasets indicate that 5hmC accumulates in ${\mathrm{\mathbf{A}}\underline{C}G}$ contexts, even though our *in vitro* data indicate that 5hmC in ${\mathrm{\mathbf{A}}\underline{C}G}$ contexts is also the best substrate for further oxidation. If TET intrinsic preferences help shape the hydroxymethylome in these cell types, these data suggest that there may be additional biological mechanisms in cellular settings that favor 5hmC persistence after generation, impeding its further oxidation. Cellular 5hmC levels likely reflect a balance between factors including catalytic turnover, replication-dependent dilution, the local chromatin environment, and TET expression, recruitment, and residence time. The enrichment of 5hmC at ACG sites *in vivo* should therefore be interpreted as a steady-state outcome rather than a direct readout of a single catalytic step. Nevertheless, these results are consistent with the observation that 5hmC, when introduced, can be a very stable modification. For example, in a mouse model, isotopically labeled 5hmC introduced early in development has been shown to remain detectable late into adulthood [12]. The results are also intriguing in the setting of recent observations that WCGW (W = A/T) sites are the most likely to lose methylation in cancer [59], suggesting that some sites might be more susceptible to DNA demethylation by both active TET-dependent and passive replication-dependent means [60]. By contrast, the prevalence of more GC-rich DNA in CpG islands, where TET enrichment has been noted, could reflect a higher barrier to activation at particular genomic sites [61, 62]. Taken together, the fact that ${\mathrm{\mathbf{A}}\underline{C}G}$ contexts, and not ${\mathrm{\mathbf{G}}\underline{C}G}$ contexts, show higher levels of 5hmC supports a model in which TET preferences for efficient 5hmC generation, rather than less efficient 5hmC depletion, could be a more important driving force in establishing the stable hydroxymethylome.

### Open questions on TET oxidation preferences

While our approach newly parses 5hmC generation from 5hmC depletion, aspects of the complex TET-mediated reaction remain open. For one, our analysis of 5hmC depletion was based on an aggregate view of product formation of 5fC and 5caC together. Our approach can be rationalized biologically by the fact that the 5hmC to 5fC transition is the key step that distinguishes the replication-dependent and replication-independent DNA demethylation pathways. Emerging studies, however, suggest potentially distinct roles for 5fC relative to 5caC [63–65], which could support added work trying to further separate 5fC and 5caC generation. Second, although our approach could simultaneously examine CpGs on both strands of duplex DNA, we could not directly evaluate how oxidation of one strand could impact the other strand. While prior data using model oligonucleotide substrates found that the oxidation status of one CpG has minimal influence on the oxidation of its partner CpG on the opposite strand [28], the added complexity associated with sequence context differences was not considered. Further studies to parse the effects of sequence context from those of opposite-strand dynamics are warranted to achieve a more complete understanding of substrate handling by TET. Third, extending future sequence preference analyses to variants of TET enzymes could offer a more complete picture. As our primary interest here was to characterize the core catalytic behavior, we focused on the highly active and well-studied hTET2-CS catalytic domain lacking the low-complexity insert [15, 16, 26, 27, 39], which enabled robust comparison across sequence contexts ranging from very poor to very good substrates. *In vivo*, TET2 interactions with partners like the DNA-binding CXXC domain of IDAX [66, 67] or post-translational modifications could have a role in modulating enzymatic activity [68–71], as could the low-complexity insert or non-catalytic domains of TET. Additionally, TET2 coexists alongside TET1 and TET3, with certain isoforms predominating in different cell types and at different developmental times. As earlier comparisons of TET family members have suggested some shared preferences for ${\mathrm{\mathbf{A}}\underline{C}G}$ contexts in 5fC/5caC generation [17, 18], added studies will be useful to further dissect the contributions of features intrinsic to the substrate (e.g. base flipping propensity) versus enzymatic determinants in dictating specificity. Indeed, given that the biological systems involve three enzymes (TET1, TET2, and TET3) reacting with three different substrates (5mC, 5hmC, and 5fC) across both CpGs of a dyad in duplex DNA, our study highlights how dissecting complexity will require further coordinated *in vitro* and cell-based studies to reveal the underlying principles governing DNA methylation dynamics.

## Supplementary Material

gkag956_Supplemental_Files

## Data Availability

NGS datasets generated or newly reanalyzed in this study are available in the GEO at https://www.ncbi.nlm.nih.gov/geo/ under the following accession numbers: [GSE319425; GSE47966 (GSM1173783, GSM1173794, GSM1173795, GSM1173779); GSE63137 (GSM1541958, GSM1541959); GSE116016 (GSM3207181, GSM3207182, GSM3207185, GSM3207186); and GSE48519 (GSM1180306, GSM1180307, GSM1180308, GSM1180315, GSM1180316)]. All original Python scripts used for analyses in this paper are publicly available in GitHub at https://github.com/erlitzkin/TET-seq-context-prefs (DOI: 10.5281/zenodo.18765450).

## Notes

Present address: Cummings School of Veterinary Medicine, Tufts University, North Grafton, MA 01536, United States

## References

[1] Bird A . The dinucleotide CG as a genomic signalling module. J Mol Biol. 2011;409:47–53. 10.1016/j.jmb.2011.01.056 21295585

[2] Tahiliani M, Koh KP, Shen Y et al. Conversion of 5-methylcytosine to 5-hydroxymethylcytosine in mammalian DNA by MLL partner TET1. Science. 2009;324:930–5. 10.1126/science.1170116 PMC2715015 19372391

[3] Ito S, Shen L, Dai Q et al. TET proteins can convert 5-methylcytosine to 5-formylcytosine and 5-carboxylcytosine. Science. 2011;333:1300–3. 10.1126/science.1210597 PMC3495246 21778364

[4] He YF, Li BZ, Li Z et al. TET-mediated formation of 5-carboxylcytosine and its excision by TDG in mammalian DNA. Science. 2011;333:1303–7. 10.1126/science.1210944 PMC3462231 21817016

[5] Seiler CL, Fernandez J, Koerperich Z et al. Maintenance DNA methyltransferase activity in the presence of oxidized forms of 5-methylcytosine: structural basis for ten-eleven translocation-mediated DNA demethylation. Biochemistry. 2018;57:6061–9. 10.1021/acs.biochem.8b00683 PMC6310613 30230311

[6] Maiti A, Drohat AC. Thymine DNA glycosylase can rapidly excise 5-formylcytosine and 5-carboxylcytosine: potential implications for active demethylation of CpG sites. J Biol Chem. 2011;286:35334–8. 10.1074/jbc.C111.284620 PMC3195571 21862836

[7] Nabel CS, Jia H, Ye Y et al. AID/APOBEC deaminases disfavor modified cytosines implicated in DNA demethylation. Nat Chem Biol. 2012;8:751–8. 10.1038/nchembio.1042 PMC3427411 22772155

[8] Globisch D, Munzel M, Muller M et al. Tissue distribution of 5-hydroxymethylcytosine and search for active demethylation intermediates. PLoS One. 2010;5:e15367. 10.1371/journal.pone.0015367 PMC3009720 21203455

[9] Münzel M, Globisch D, Brückl T et al. Quantification of the sixth DNA base hydroxymethylcytosine in the brain. Angew Chem Int Ed Engl. 2010;49:5375–7. 10.1002/anie.201002033 20583021

[10] Lee SM, Goldberg DC, Cloud C et al. A ternary-code DNA methylome atlas of mouse tissues. Genome Biol. 2025;26:343. 10.1186/s13059-025-03808-y PMC12506328 41057935

[11] Cui X, Nie J, Ku J et al. A human tissue map of 5-hydroxymethylcytosines exhibits tissue specificity through gene and enhancer modulation. Nat Commun. 2020;11:6161. 10.1038/s41467-020-20001-w PMC7710742 33268789

[12] Bachman M, Uribe-Lewis S, Yang X et al. 5-hydroxymethylcytosine is a predominantly stable DNA modification. Nature Chem. 2014;6:1049–55. 10.1038/nchem.2064 PMC4382525 25411882

[13] Booth MJ, Branco MR, Ficz G et al. Quantitative sequencing of 5-methylcytosine and 5-hydroxymethylcytosine at single-base resolution. Science. 2012;336:934–7. 10.1126/science.1220671 22539555

[14] Lercher L, McDonough MA, El-Sagheer AH et al. Structural insights into how 5-hydroxymethylation influences transcription factor binding. Chem Commun. 2014;50:1794–6. 10.1039/C3CC48151D 24287551

[15] Hu L, Li Z, Cheng J et al. Crystal structure of TET2-DNA complex: insight into TET-mediated 5mC oxidation. Cell. 2013;155:1545–55. 10.1016/j.cell.2013.11.020 24315485

[16] Hu L, Lu J, Cheng J et al. Structural insight into substrate preference for TET-mediated oxidation. Nature. 2015;527:118–22. 10.1038/nature15713 26524525

[17] Adam S, Bräcker J, Klingel V et al. Flanking sequences influence the activity of TET1 and TET2 methylcytosine dioxygenases and affect genomic 5hmC patterns. Commun Biol. 2022;5:92–4. 10.1038/s42003-022-03033-4 PMC8786823 35075236

[18] Ravichandran M, Rafalski D, Davies CI et al. Pronounced sequence specificity of the TET enzyme catalytic domain guides its cellular function. Sci Adv. 2022;8:eabm2427. 10.1126/sciadv.abm2427 PMC9451156 36070377

[19] Adam S, Anteneh H, Hornisch M et al. DNA sequence-dependent activity and base flipping mechanisms of DNMT1 regulate genome-wide DNA methylation. Nat Commun. 2020;11:3723–8. 10.1038/s41467-020-17531-8 PMC7381644 32709850

[20] Dukatz M, Adam S, Biswal M et al. Complex DNA sequence readout mechanisms of the DNMT3B DNA methyltransferase. Nucleic Acids Res. 2020;48:11495–509. 10.1093/nar/gkaa938 PMC7672481 33105482

[21] Mao S, Cuesta SM, Tannahill D et al. Genome-wide DNA methylation signatures are determined by DNMT3A/B sequence preferences. Biochemistry. 2020;59:2541–50. 10.1021/acs.biochem.0c00339 PMC7364778 32543182

[22] Gao L, Emperle M, Guo Y et al. Comprehensive structure-function characterization of DNMT3B and DNMT3A reveals distinctive *de novo* DNA methylation mechanisms. Nat Commun. 2020;11:3355–4. 10.1038/s41467-020-17109-4 PMC7335073 32620778

[23] Emperle M, Adam S, Kunert S et al. Mutations of R882 change flanking sequence preferences of the DNA methyltransferase DNMT3A and cellular methylation patterns. Nucleic Acids Res. 2019;47:11355–67. 10.1093/nar/gkz911 PMC6868496 31620784

[24] Mallona I, Ilie IM, Karemaker ID et al. Flanking sequence preference modulates *de novo* DNA methylation in the mouse genome. Nucleic Acids Res. 2021;49:145–57. 10.1093/nar/gkaa1168 PMC7797059 33290556

[25] Wang T, Kohli RM. Discovery of an unnatural DNA modification derived from a natural secondary metabolite. Cell Chem Biol. 2021;28:97–104. 10.1016/j.chembiol.2020.09.006 PMC7855694 33053370

[26] Liu MY, DeNizio JE, Kohli RM. Quantification of oxidized 5-methylcytosine bases and TET enzyme activity. Methods Enzymol. 2016;573:365–85. 10.1016/bs.mie.2015.12.006 PMC5125823 27372762

[27] Liu MY, Torabifard H, Crawford DJ et al. Mutations along a TET2 active site scaffold stall oxidation at 5-hydroxymethylcytosine. Nat Chem Biol. 2017;13:181–7. 10.1038/nchembio.2250 PMC5370579 27918559

[28] Crawford DJ, Liu MY, Nabel CS et al. Tet2 catalyzes stepwise 5-methylcytosine oxidation by an iterative and *de novo* mechanism. J Am Chem Soc. 2016;138:730–3. 10.1021/jacs.5b10554 PMC4762542 26734843

[29] Ito S, D’Alessio AC, Taranova OV et al. Role of tet proteins in 5mC to 5hmC conversion, ES-cell self-renewal, and inner cell mass specification. Nature. 2010;466:1129–33. 10.1038/nature09303 PMC3491567 20639862

[30] Schutsky EK, DeNizio JE, Hu P et al. Nondestructive, base-resolution sequencing of 5-hydroxymethylcytosine using a DNA deaminase. Nat Biotechnol. 2018;36:1083–90. 10.1038/nbt.4204 PMC6453757 30295673

[31] Caldwell BA, Liu MY, Prasasya RD et al. Functionally distinct roles for TET-oxidized 5-methylcytosine bases in somatic reprogramming to pluripotency. Mol Cell. 2021;81:859–69.e8. 10.1016/j.molcel.2020.11.045 PMC7897302 33352108

[32] Wang T, Loo CE, Kohli RM. Enzymatic approaches for profiling cytosine methylation and hydroxymethylation. Mol Metab. 2021;57:101314. 10.1016/j.molmet.2021.101314 PMC8829811 34375743

[33] Fabyanic EB, Hu P, Qiu Q et al. Joint single-cell profiling resolves 5mC and 5hmC and reveals their distinct gene regulatory effects. Nat Biotechnol. 2023;42:960–74. 10.1038/s41587-023-01909-2 PMC11525041 37640946

[34] Lister R, Mukamel EA, Nery JR et al. Global epigenomic reconfiguration during mammalian brain development. Science. 2013;341:1237905. 10.1126/science.1237905 PMC3785061 23828890

[35] Mo A, Mukamel EA, Davis FP et al. Epigenomic signatures of neuronal diversity in the mammalian brain. Neuron. 2015;86:1369–84. 10.1016/j.neuron.2015.05.018 PMC4499463 26087164

[36] Hon GC, Song C, Du T et al. 5mC oxidation by Tet2 modulates enhancer activity and timing of transcriptome reprogramming during differentiation. Mol Cell. 2014;56:286–97. 10.1016/j.molcel.2014.08.026 PMC4319980 25263596

[37] Goldberg DC, Fu H, Atkins D et al. KnowYourCG: facilitating base-level sparse methylome interpretation. Sci Adv. 2025;11:eadw3027. 10.1126/sciadv.adw3027 PMC12551721 41134907

[38] Rauluseviciute I, Riudavets-Puig R, Blanc-Mathieu R et al. JASPAR 2024: 20th anniversary of the open-access database of transcription factor binding profiles. Nucleic Acids Res. 2024;52:D174–82. 10.1093/nar/gkad1059 PMC10767809 37962376

[39] DeNizio JE, Liu MY, Leddin EM et al. Selectivity and promiscuity in TET-mediated oxidation of 5-methylcytosine in DNA and RNA. Biochemistry. 2019;58:411–21. 10.1021/acs.biochem.8b00912 PMC6363868 30387995

[40] Wang T, Fowler JM, Liu L et al. Direct enzymatic sequencing of 5-methylcytosine at single-base resolution. Nat Chem Biol. 2023;19:1004–12. 10.1038/s41589-023-01318-1 PMC10763687 37322153

[41] Loo CE, Fowler JM, Zhu H et al. Sparse sequencing permits accurate and efficient quantification of genome-wide cytosine modification levels. Genom Bioinform. 2026;8:lqag082. 10.1093/nargab/lqag082 PMC13408044 42523907

[42] O’Neil LL, Wiest O. Structures and energetics of base flipping of the thymine dimer depend on DNA sequence. J Phys Chem B. 2008;112:4113–22. 10.1021/jp7102935 18335922

[43] Várnai P, Lavery R. Base flipping in DNA: pathways and energetics studied with molecular dynamic simulations. J Am Chem Soc. 2002;124:7272–3. 10.1021/ja025980x 12071727

[44] Yakovchuk P, Protozanova E, Frank-Kamenetskii MD. Base-stacking and base-pairing contributions into thermal stability of the DNA double helix. Nucleic Acids Res. 2006;34:564–74. 10.1093/nar/gkj454 PMC1360284 16449200

[45] Zhang T, Zhang C, Dong Z et al. Determination of base binding strength and base stacking interaction of DNA duplex using atomic force microscope. Sci Rep. 2015;5:9143. 10.1038/srep09143 PMC4360479 25772017

[46] Viader-Godoy X, Manosas M, Ritort F. Stacking correlation length in single-stranded DNA. Nucleic Acids Res. 2024;52:13243–54. 10.1093/nar/gkae934 PMC11602145 39460618

[47] Plumridge A, Meisburger SP, Andresen K et al. The impact of base stacking on the conformations and electrostatics of single-stranded DNA. Nucleic Acids Res. 2017;45:3932–43. 10.1093/nar/gkx140 PMC5397193 28334825

[48] Ke C, Humeniuk M, S-Gracz H et al. Direct measurements of base stacking interactions in DNA by single-molecule atomic-force spectroscopy. Phys Rev Lett. 2007;99:018302. 10.1103/PhysRevLett.99.018302 17678193

[49] O’Neil LL, Wiest O. Sequence dependence in base flipping: experimental and computational studies. Org Biomol Chem. 2008;6:485–92. 10.1039/B713318A 18219418

[50] Banavali NK, MacKerell AD. Free energy and structural pathways of base flipping in a DNA GCGC containing sequence. J Mol Biol. 2002;319:141–60. 10.1016/S0022-2836(02)00194-8 12051942

[51] Prasasya RD, Caldwell BA, Liu Z et al. Iterative oxidation by TET1 is required for reprogramming of imprinting control regions and patterning of mouse sperm hypomethylated regions. Dev Cell. 2024;59:1010–27. 10.1016/j.devcel.2024.02.012 PMC11042979 38569549

[52] DeNizio JE, Dow BJ, Serrano JC et al. TET-TDG active DNA demethylation at CpG and non-CpG sites. J Mol Biol. 2021;433:166877. 10.1016/j.jmb.2021.166877 PMC8005466 33561435

[53] Ren R, Horton JR, Hong S et al. Recent advances on DNA base flipping: a general mechanism for writing, reading, and erasing DNA modifications. Adv Exp Med Biol. 2022;1389:295–315. 10.1007/978-3-031-11454-0 10.1007/978-3-031-11454-0_12 PMC9711002 36350515

[54] Ayadi L, Jourdan M, Coulombeau C et al. Experimental and theoretical studies of the conformational perturbations induced by an abasic site. J Biomol Struct Dyn. 1999;17:245–57. 10.1080/07391102.1999.10508357 10563574

[55] Folta-Stogniew E, Russu IM. Sequence dependence of base-pair opening in a DNA dodecamer containing the CACA/GTGT sequence motif. Biochemistry. 1994;33:11016–24. 10.1021/bi00202a022 8086418

[56] Tamanaha E, Guan S, Marks K et al. Distributive processing by the iron(II)/Î±-ketoglutarate-dependent catalytic domains of the TET enzymes is consistent with epigenetic roles for oxidized 5-methylcytosine bases. J Am Chem Soc. 2016;138:9345–8. 10.1021/jacs.6b03243 27362828

[57] Punnoose JA, Kam C, Cole D et al. Quantifying the base-stacking energy in nucleic acids using a single-molecule force clamp assay. Biophys J. 2024;123:459a. 10.1016/j.bpj.2023.11.2787

[58] Szulik MW, Pallan PS, Nocek B et al. Differential stabilities and sequence-dependent base pair opening dynamics of Watson–Crick base pairs with 5-hydroxymethylcytosine, 5-formylcytosine, or 5-carboxylcytosine. Biochemistry. 2015;54:1294–305. 10.1021/bi501534x PMC4325598 25632825

[59] Zhou W, Dinh HQ, Ramjan Z et al. DNA methylation loss in late-replicating domains is linked to mitotic cell division. Nat Genet. 2018;50:591–602. 10.1038/s41588-018-0073-4 PMC5893360 29610480

[60] López-Moyado IF, Hernández-Espinosa L, Angel JC et al. Flanking DNA sequences determine DNA methylation maintenance in proliferation, cancer and aging. bioRxiv, 10.64898/2026.04.09.717557, 11 April 2026, preprint: not peer reviewed.

[61] Williams K, Christensen J, Pedersen MT et al. TET1 and hydroxymethylcytosine in transcription and DNA methylation fidelity. Nature. 2011;473:343–8. 10.1038/nature10066 PMC3408592 21490601

[62] Williams K, Christensen J, Helin K. DNA methylation: TET proteins-guardians of CpG islands?. EMBO Rep. 2011;13:28–35. 10.1038/embor.2011.233 PMC3246258 22157888

[63] Bachman M, Uribe-Lewis S, Yang X et al. 5-formylcytosine can be a stable DNA modification in mammals. Nat Chem Biol. 2015;11:555–7. 10.1038/nchembio.1848 PMC5486442 26098680

[64] Iurlaro M, McInroy GR, Burgess HE et al. *In vivo* genome-wide profiling reveals a tissue-specific role for 5-formylcytosine. Genome Biol. 2016;17:141–5. 10.1186/s13059-016-1001-5 PMC4928330 27356509

[65] Iurlaro M, Ficz G, Oxley D et al. A screen for hydroxymethylcytosine and formylcytosine binding proteins suggests functions in transcription and chromatin regulation. Genome Biol. 2013;14:R119. 10.1186/gb-2013-14-10-r119 PMC4014808 24156278

[66] Ko M, An J, Bandukwala HS et al. Modulation of TET2 expression and 5-methylcytosine oxidation by the CXXC domain protein IDAX. Nature. 2013;497:122–6. 10.1038/nature12052 PMC3643997 23563267

[67] Dunican DS, Pennings S, Meehan RR. The CXXC-TET bridge–mind the methylation gap!. Cell Res. 2013;23:973–4. 10.1038/cr.2013.71 PMC3731560 23711677

[68] Zhang X, Han B, Shao Z et al. Auto-suppression of tet dioxygenases protects the mouse oocyte genome from oxidative demethylation. Nat Struct Mol Biol. 2024;31:42–53. 10.1038/s41594-023-01125-1 38177668

[69] Zhang YW, Wang Z, Xie W et al. Acetylation enhances TET2 function in protecting against abnormal DNA methylation during oxidative stress. Mol Cell. 2017;65:323–35. 10.1016/j.molcel.2016.12.013 PMC5260804 28107650

[70] Kundu A, Shelar S, Ghosh AP et al. 14-3-3 proteins protect AMPK-phosphorylated ten-eleven translocation-2 (TET2) from PP2A-mediated dephosphorylation. J Biol Chem. 2020;295:1754–66. 10.1074/jbc.RA119.011089 PMC7008385 31901078

[71] Lejart A, Zentout S, Chapuis C et al. The N-terminal domain of TET1 promotes the formation of dense chromatin regions refractory to transcription. Chromosoma. 2022;131:47–58. 10.1007/s00412-022-00769-0 35235010

