# Supplementary material for: Genomic sequence context differentially impacts stepwise oxidation by TET enzymes to shape DNA hydroxymethylation landscapes
Source: Nucleic Acids Res. 2026 Oct 13;54(19):gkag956. doi: 10.1093/nar/gkag956 (PMC13659243; doi:10.1093/nar/gkag956)
Supplement: Supplementary file 1 [file gkag956_supplemental_files.zip › SI-Clean.pdf]

SUPPORTING INFORMATION

Genomic sequence context differentially impacts stepwise oxidation by TET enzymes to shape DNA hydroxymethylation landscapes

Noa Erlitzki[1,2], Christian E. Loo[1], Johanna M. Fowler[2,3], Wanding Zhou[4,5], Rahul M. Kohli[2,*]

1 Graduate Group in Biochemistry, Biophysics, and Chemical Biology, Perelman School of Medicine, University of Pennsylvania, Philadelphia, PA, 19104, USA
2 Department of Medicine, Perelman School of Medicine, University of Pennsylvania, Philadelphia, PA, 19104, USA
3 Present address: Cummings School of Veterinary Medicine, Tufts University, North Grafton, MA, 01536, USA
4 Center for Computational and Genomic Medicine, The Children's Hospital of Philadelphia, Philadelphia, PA, 19104, USA
5 Department of Pathology and Laboratory Medicine, University of Pennsylvania, Philadelphia, PA, 19104, USA

* To whom correspondence should be addressed. Email: rkohli@pennmedicine.upenn.edu

Supplemental Figures and Tables .... Page S2

Figure S1. Methods for resolving modified cytosine bases .... Page S2
Figure S2. Characteristics of the context-diverse methylated substrates .... Page S3
Figure S3. Flow diagram of data exclusion criteria and attrition of CpG sites.... Page S4
Figure S4. Modified adapters with unnatural cytosines can support dual-sequencing pipeline .... Page S5
Figure S5. Distribution of $EC50_1$ and $EC50_2$ values across all CpG sites.......... Page S6
Figure S6. Influence of CpG position and local density on TET reactivity .... Page S7
Figure S7. Alternative analyses show 5hmC generation and depletion depend on the bases flanking the CpG site .... Page S8
Figure S8. Relative reaction progress at ACGT, CCGG, GCGC, and TCGA sites within double-stranded, single-stranded, or mismatch DNA substrates .... Page S9
Figure S9. The presence of a 5mC:T mismatch does not impede DNA duplex formation .... Page S10
Figure S10. Sequence context preferences for TET oxidation of CpGs within the methylated KvDMR-containing substrate .... Page S11
Figure S11. Kinetic analysis of sequence context preferences .... Page S12
Figure S12. 5hmC accumulation tracks CpG flanking-base composition at transcription factor binding sites *in vivo* .... Page S13
Supplementary Table 1. Comparative analysis of the 5hmC generation ($EC50_1$) and depletion ($EC50_2$) for CpG sites in different "4-mer" sequence contexts .... Page S14
Supplementary Table 2. TET reactivity parameters extracted from curve fits utilizing the KvDMR-containing methylated substrate .... Page S15

SUPPLEMENTAL FIGURES AND TABLES

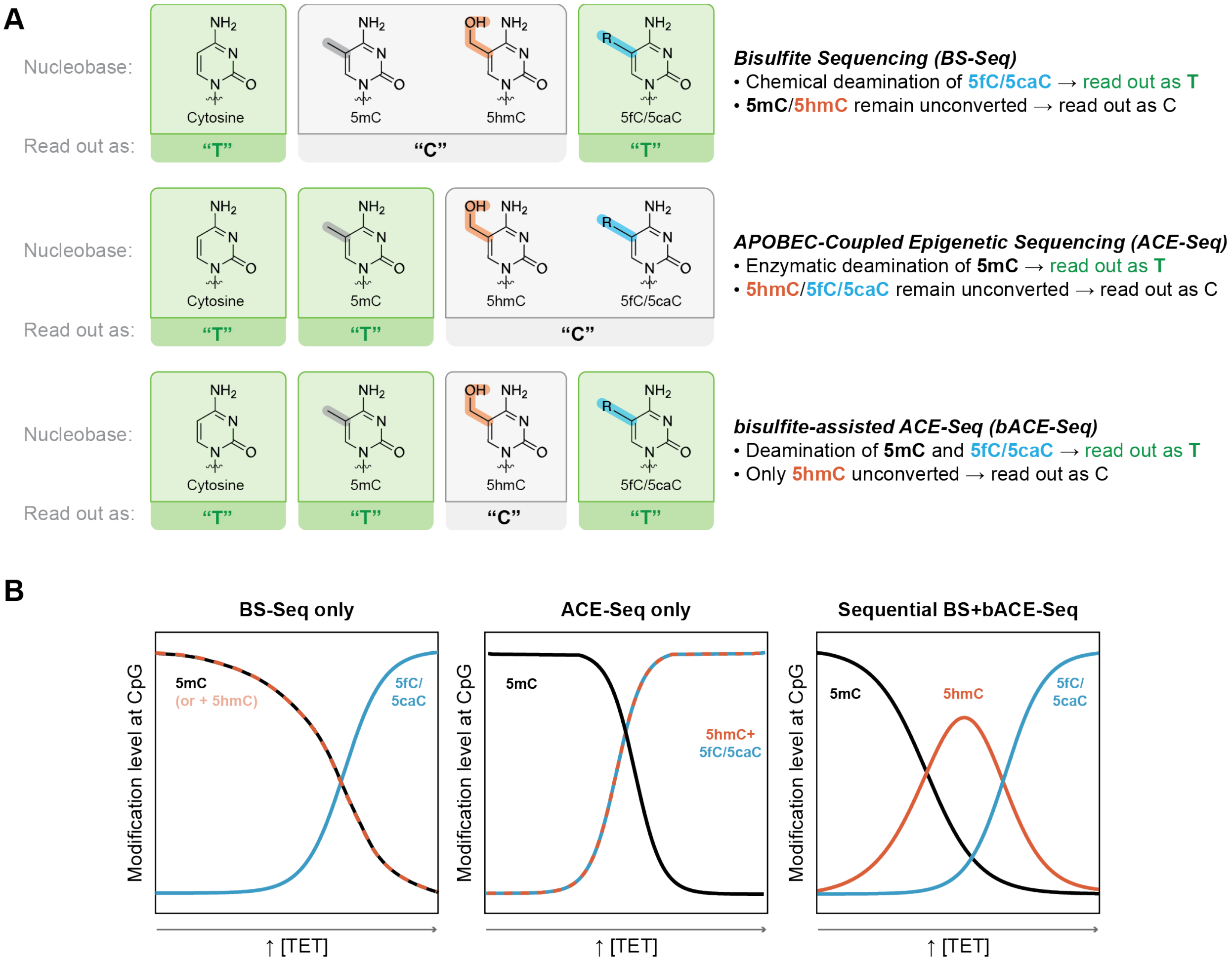

Figure S1. Methods for resolving modified cytosine bases. (**A**) Shown are unmodified cytosine, 5mC, 5hmC and 5fC/5caC readouts from chemical (BS-Seq), enzymatic (ACE-Seq), and mixed chemical/enzymatic (bACE-Seq) methods. (**B**) Schematic depicts results that can be obtained when starting from DNA modified with 5mCpG (or 5hmCpG), with the sequential BS/bACE-Seq pipeline allowing for monitoring of 5hmC accumulation and its decay to 5fC/5caC from the same sample.

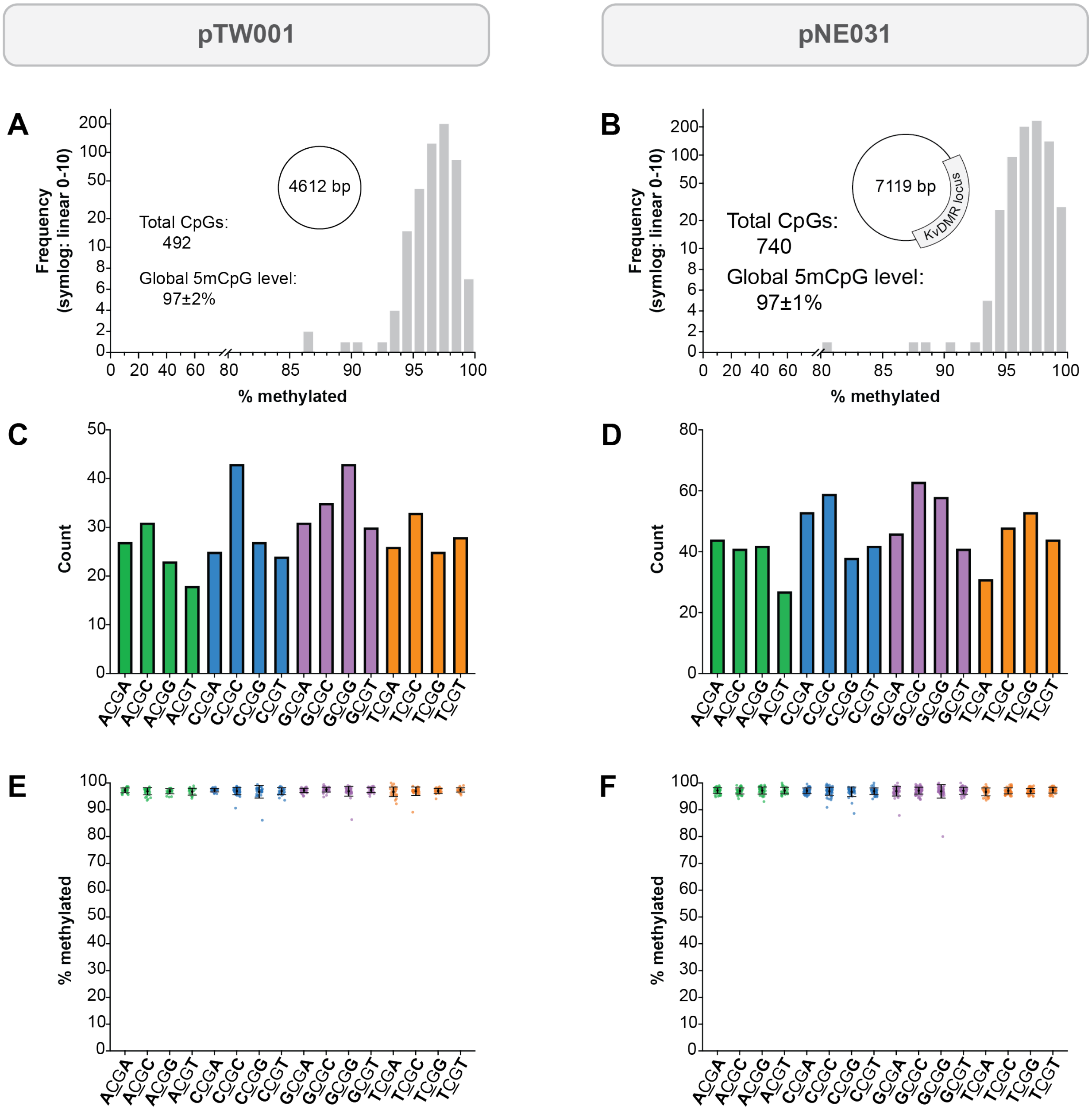

Figure S2. Characteristics of the context-diverse methylated substrates. (A, B) The total number of CpG sites in each plasmid and the overall methylation levels are shown superimposed on frequency distribution plots of per-CpG methylation levels as determined by BS-seq. **(C, D)** Number of CpG sites analyzed in each 4-mer context for each substrate. **(E, F)** The initial methylation levels of CpG sites are shown grouped by 4-mer context. Individual values are shown for each CpG site with mean ± SD plotted.

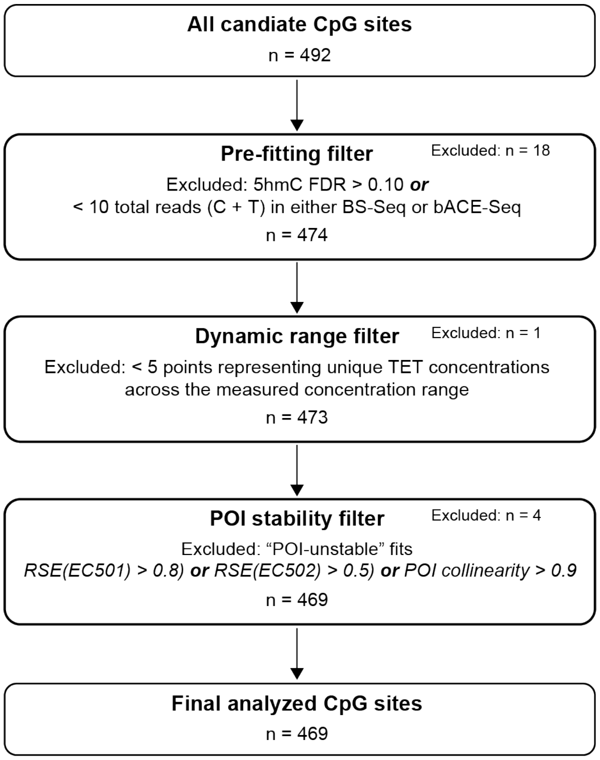

Figure S3. Flow diagram of data exclusion criteria and attrition of CpG sites. Shown is the filtering process to assure data quality for the context-diverse methylated substrate**.** FDR = false detection rate, POI = parameter of interest, RSE = relative standard error.

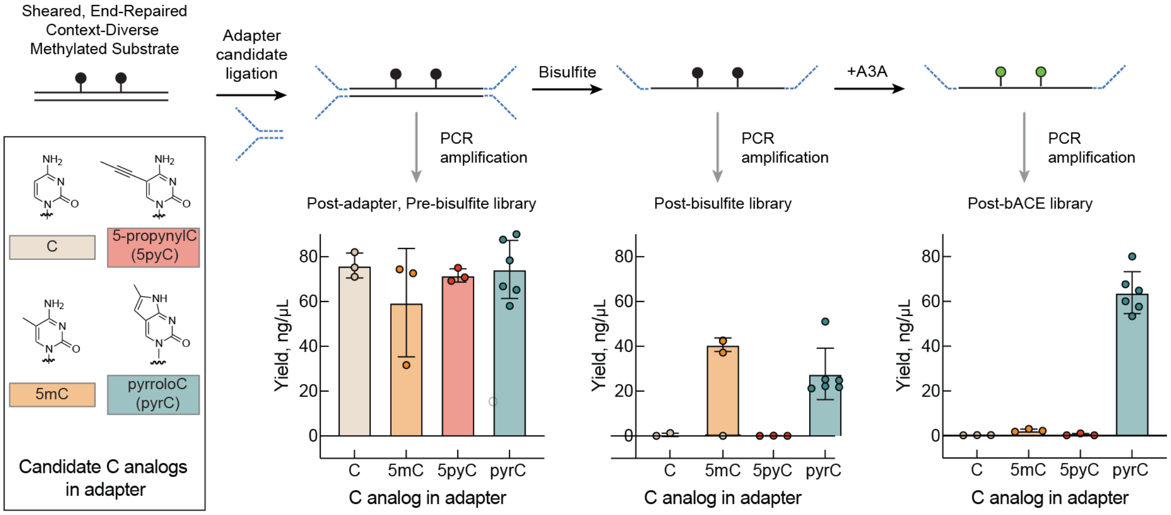

Figure S4. Modified adapters with unnatural cytosines can support dual-sequencing pipeline. Sheared DNA from the context-diverse methylated substrate was ligated to sequencing adapters where all cytosines are replaced with either C, 5mC, 5-propynylcytosine (5pyC), or pyrrolocytosine (pyrC). The samples were subjected to bisulfite sequencing, and half of the resulting bisulfite-treated DNA was then subjected to enzymatic deamination. Library construction was attempted for each sample before bisulfite treatment, after bisulfite, or after bisulfite and A3A using primers that should anneal to the adapter sequences. Shown are the quantified yields of the potential libraries after PCR amplification with 12, 20, and 22 cycles respectively (accounting for sample loss to degradation from bisulfite or with purification steps). Individual values are shown (n = 3-6 per condition) with mean ± SD plotted. The results demonstrate that samples with C-containing adapters permit amplification before bisulfite, but not after. 5mC-containing adapters support amplification after chemical but not enzymatic deamination, while samples with 5pyC-containing adapters are not amplifiable after bisulfite treatment. For samples with pyrC-containing adapters, library generation is supported after both bisulfite and subsequent enzymatic deamination.

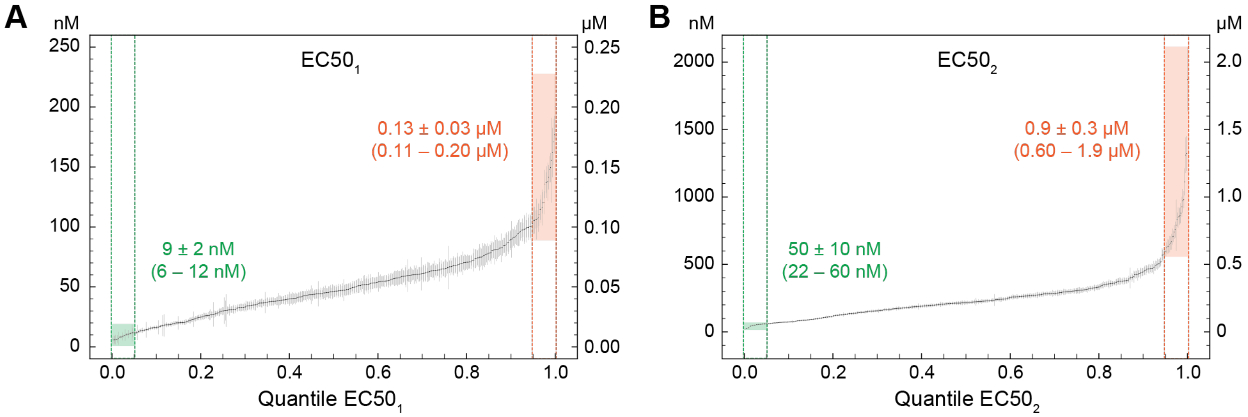

Figure S5. Distribution of $EC50_1$ and $EC50_2$ values across all CpG sites. For the context-diverse methylated substrate, absolute values of $EC50_1$ (**A**) and $EC50_2$ (**B**) are plotted in order, defined as the quantile from 0 to 1. The mean ± SD and range of CpG sites in the bottom 5th (most reactive) and top 5th quantiles (least reactive) are shown in green and orange, respectively.

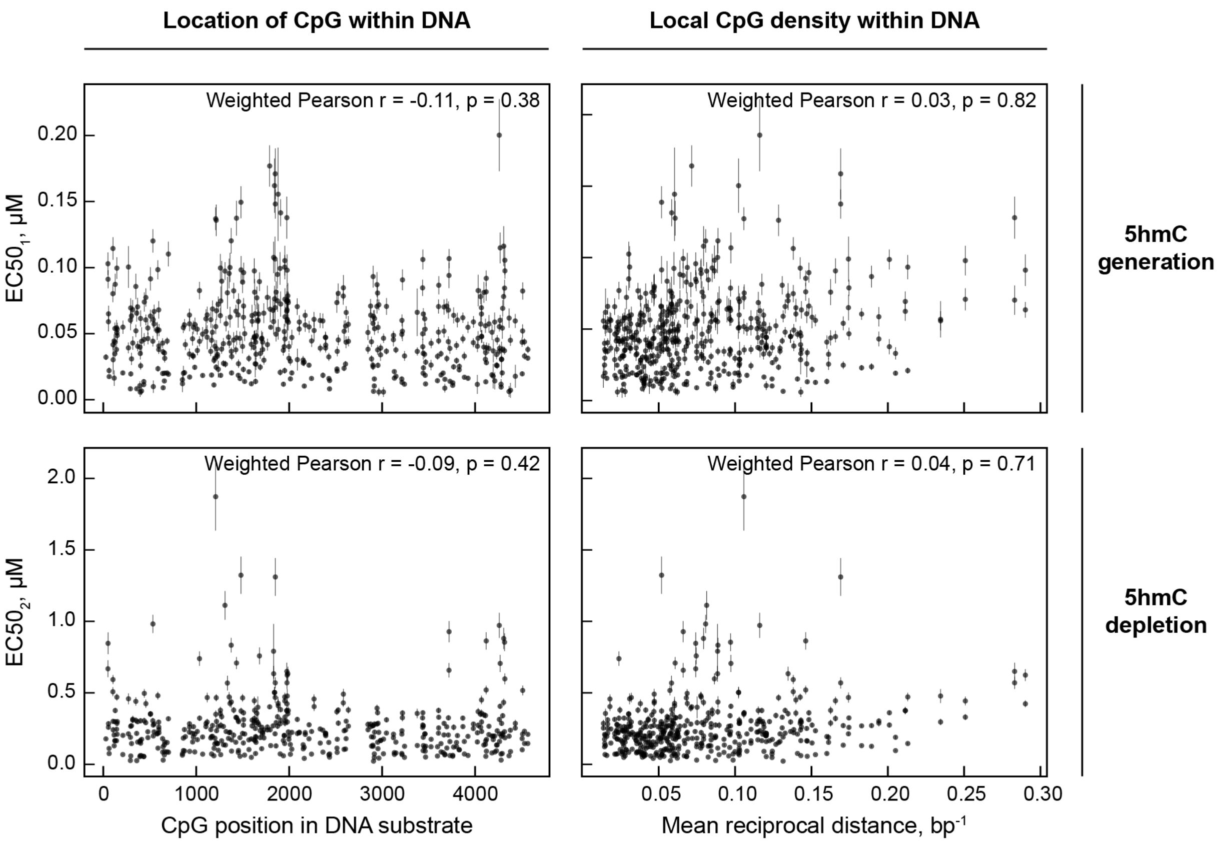

Figure S6. Influence of CpG position and local density on TET reactivity. Neither the positioning of the CpG site within the DNA substrate nor the local density of CpG sites strongly correlates with the observed efficiency of 5hmC generation or depletion. Left: $EC50_1$ (top) and $EC50_2$ values (bottom) for individual CpG sites are plotted in the order each site appears in the context-diverse methylated DNA substrate (shown as a linearized view relative to position 1 of the linearized plasmid DNA). Right: The same $EC50_1$ and $EC50_2$ values for individual CpG sites are plotted against the local density of CpG sites, given as the mean reciprocal distance (MRD) to the five same-strand, nearest-neighbor CpG sites on either side of each CpG. MRD was computed by calculating the distance in bp to each of the five closest CpG sites on the same strand as a given CpG, calculating the reciprocal value of each distance, and then taking the mean of the five reciprocal distances such that higher values of MRD correspond to higher local CpG density.

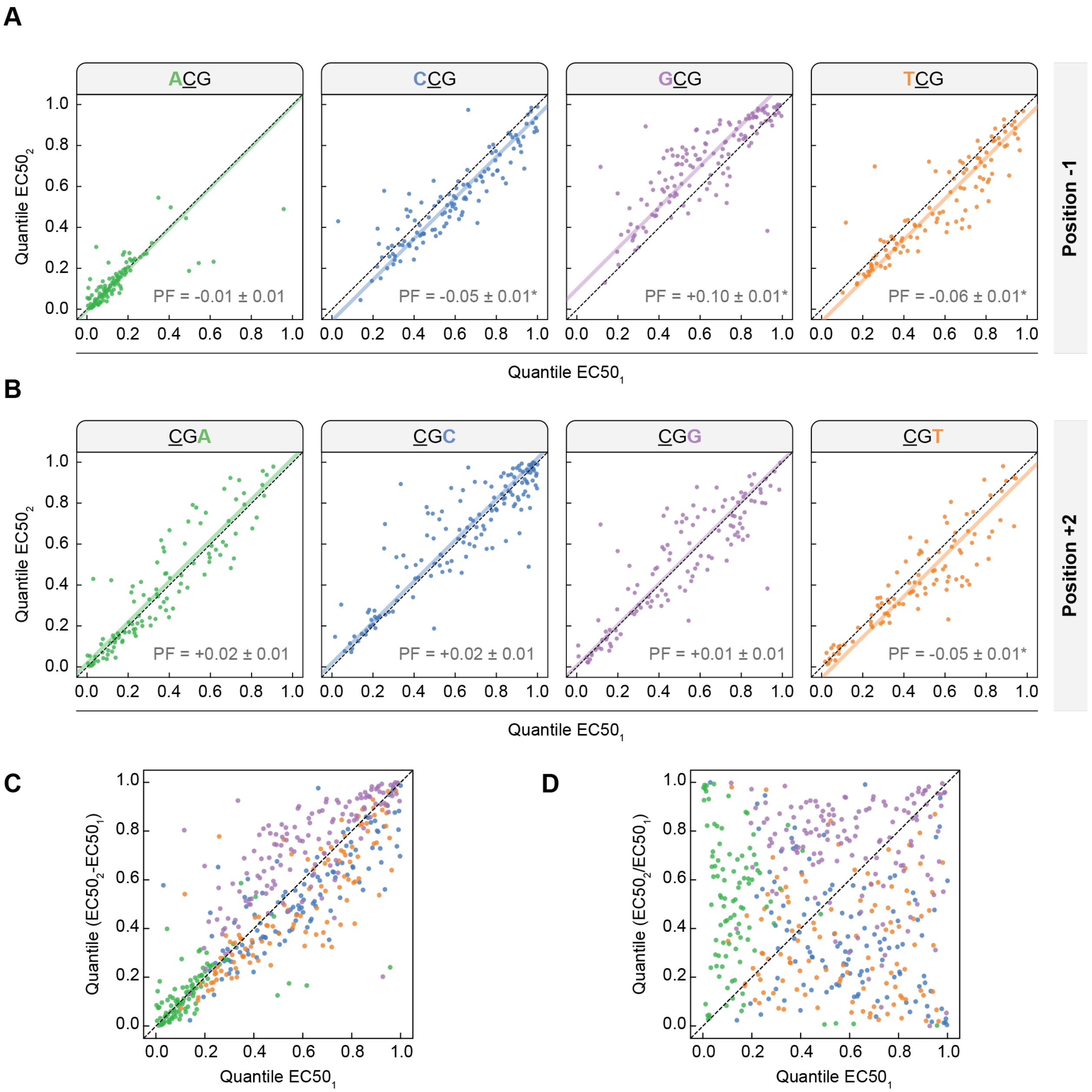

Figure S7. Alternative analyses show 5hmC generation and depletion depend on the bases flanking the CpG site. (**A-B**) Shown are the comparison scatter plots in which each CpG site is represented by a point with coordinates corresponding to its rank orders among all CpGs for $EC50_1$ and $EC50_2$. The plot is identical to **Figure 3D** except that data points not corresponding to the sequence context of interest are removed for improved clarity, and the best-fit line with slope =1 is shown to highlight the determined persistence factor (PF), determined by quantified by fitting a linear model with the slope fixed to 1 to test for significant systematic offsets of the data from the line of identity. The resulting slope with ± 95% CI are provided, with * denoting $p < 0.0005$ for deviation from 0. (**C**) Shown is a comparison scatter plots comparing the relative ranking of the $EC50_1$ with the difference between $EC50_2$ with $EC50_1$. Each individual data point is a single CpG with coloring based on the identity of the -1 position as in (A). (**D**) Shown is a comparison scatter plots comparing the relative ranking of the $EC50_1$ with the ratio of $EC50_2$ with $EC50_1$. Each individual data point is a single CpG with coloring based on the identity of the -1 position as in (A).

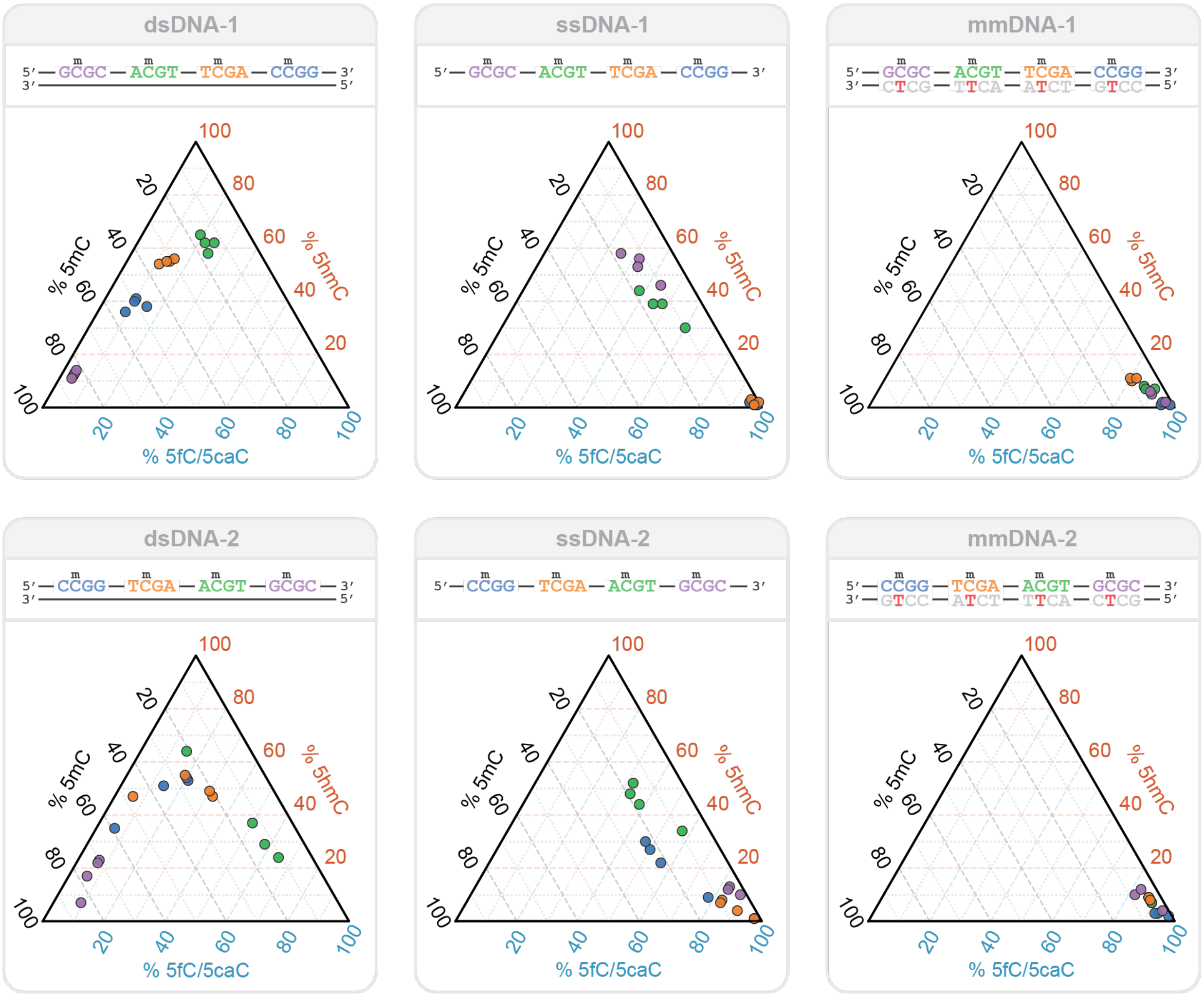

Figure S8. Relative reaction progress at ACGT, CCGG, GCGC, and TCGA sites within double-stranded, single-stranded, or mismatch DNA substrates. Ternary plots show the reaction progress from 5mC to 5hmC to 5fC/5caC at each of the tested CpG sites following reaction with 0.5 μM TET for 1 hour. On the ternary plot, each data point shows the value of unmodified C, 5mC and 5hmC, with the three values summed to 100%. Points on the plot are colored by sequence context. Points that are the same color represent independent replicate samples (n = 3-4 per substrate). The pie charts in Figure 5C-D show averages of these replicates.

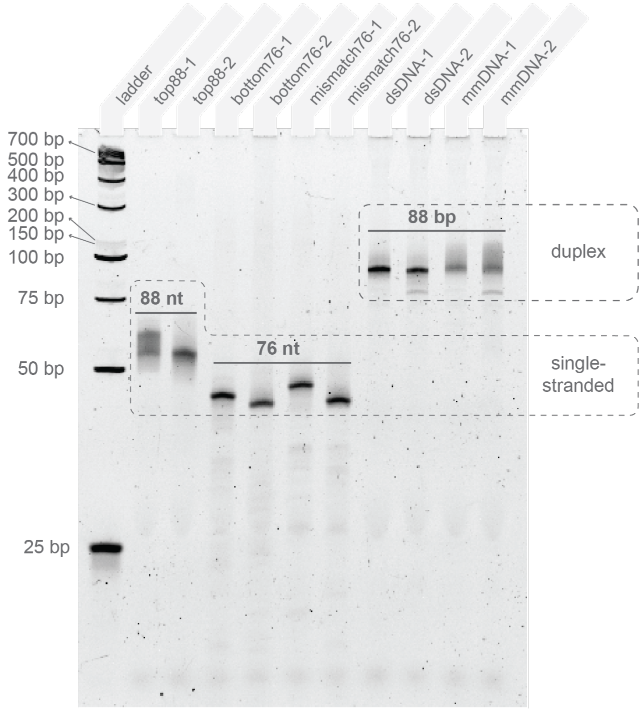

Figure S9. The presence of a 5mC:T mismatch does not impede DNA duplex formation. Single-stranded and duplexed oligos were visualized on a non-denaturing polyacrylamide fel (12% acrylamide/bis 19:1, post-stained with SYBR Gold). 25 ng of each sample was loaded onto the gel. The ladder shows dsDNA of varying size as noted.

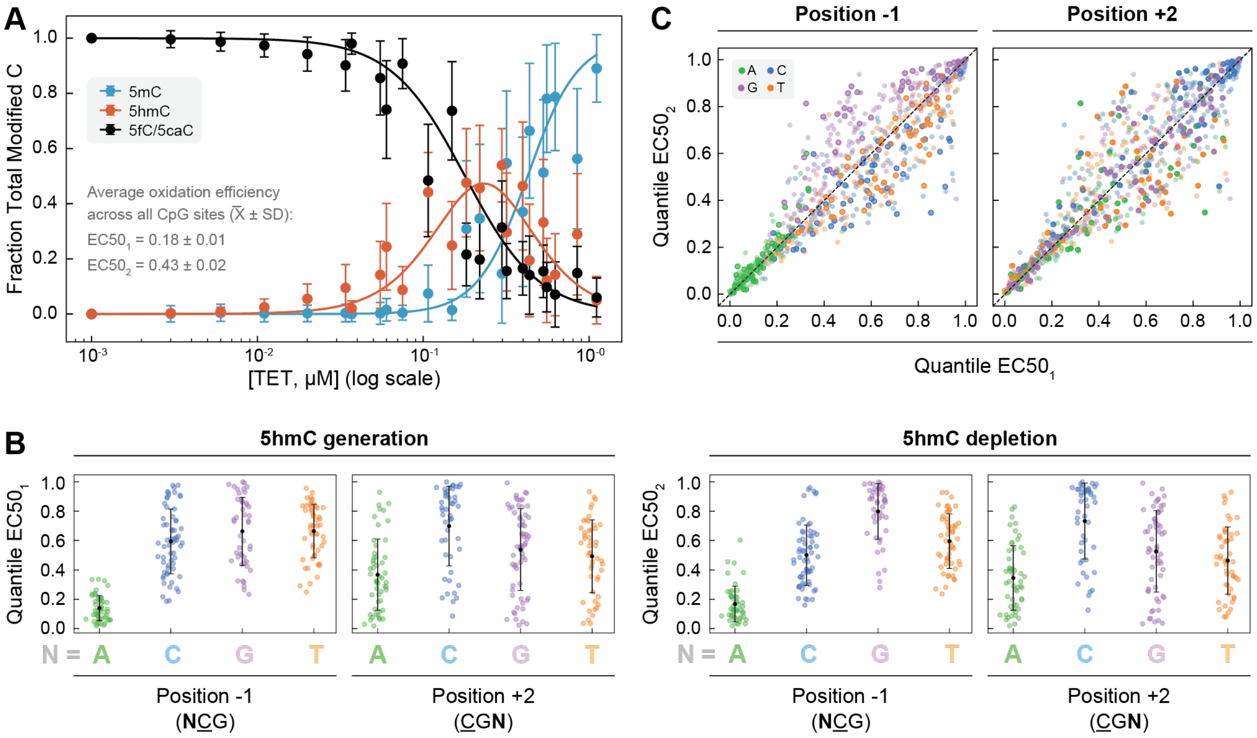

Figure S10. Sequence context preferences for TET oxidation of CpGs within the methylated KvDMR-containing substrate locus. (**A**) Titration curves of aggregate data across all CpGs in the methylated KvDMR-containing substrate, with points plotted as mean fraction total modified cytosine ± SD. (**B**) Relative efficiency of 5hmC generation and depletion at CpGs specifically within the KvDMR locus grouped by base identity at the -1 position (left-side panels) or +2 position (right-side panels). Per-group mean ± SD values are overlayed in black. (**C**) Comparison scatter plots of per-CpG correlated 5hmC generation and depletion efficiencies relative to all other CpGs. Points representing CpG sites within the KvDMR locus are emphasized as larger circles with solid outlines and are shown against a background of all other (non-KvDMR) CpG sites in the substrate (semi-opaque, non-outlined circles), demonstrating that the KvDMR-derived sequence behaves similarly to the reference sequence. Individual data points are shown, with mean ± SD plotted.

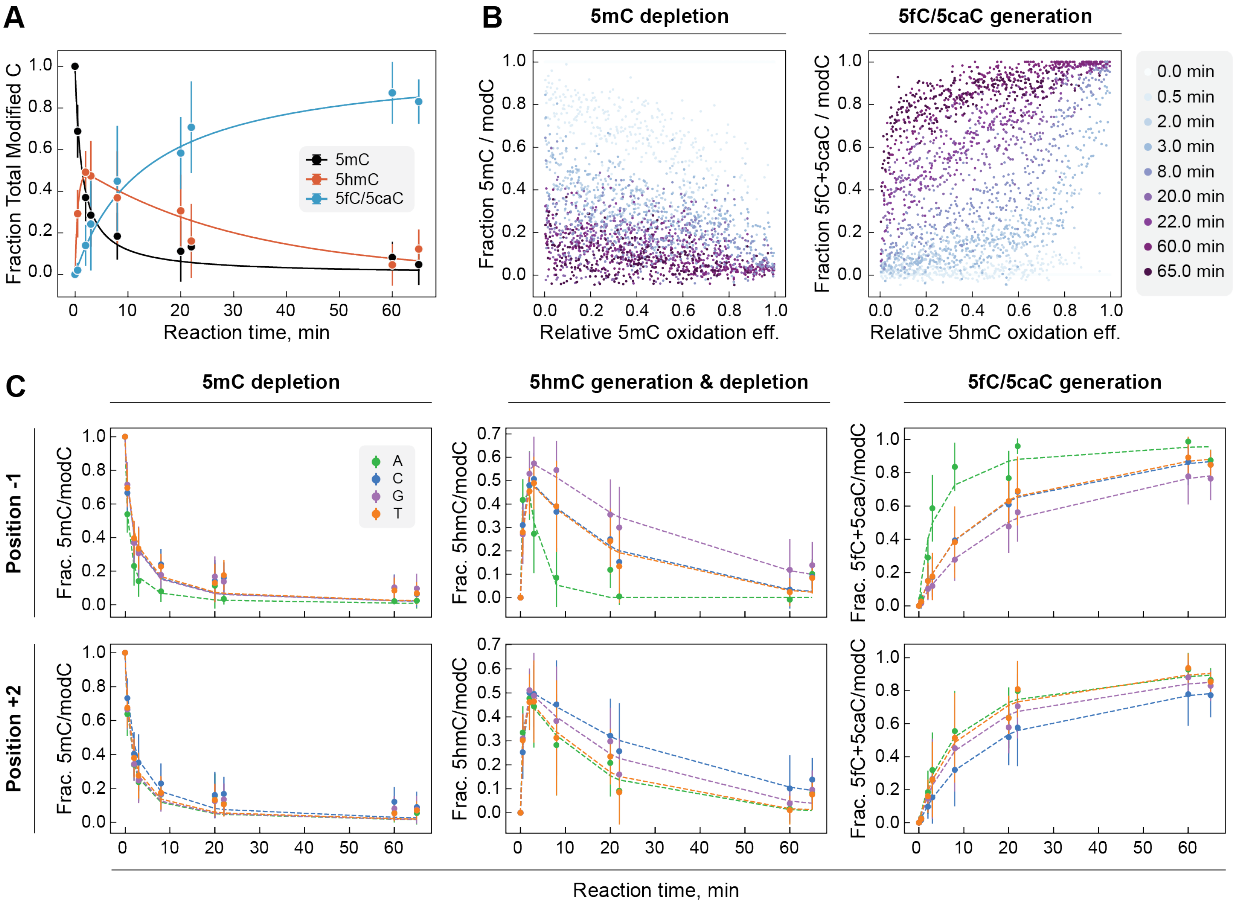

Figure S11. Kinetic analysis of sequence context preferences. Oxidation of methylated-CpG sites in the context-diverse methylated substrate in the presence of 1.1 μM TET were measured as a function of time. (**A**) Reaction progress of stepwise TET oxidation as a function of time, plotted as aggregate data (mean ± SD) across all CpGs in the substrate. Curves are splines to guide visualization only. (**B**) Scatter plots of individual CpGs comparing the time-dependent depletion of 5mC (left) or formation of 5fC/5caC (right) to the relative efficiency of each step as determined by the titration assays, where "relative 5mC oxidation efficiency" = |1 - (quantile $EC50_1$)| and "relative 5hmC oxidation efficiency" = |1 - (quantile $EC50_2$)| such that efficiency increases going to the right. CpG sites that were more efficient substrates in the titration assays were also CpG sites at which 5mC was depleted or 5fC/5caC was formed more rapidly in this time-course assay. (**C**) Reaction progress shown separately for 5mC depletion, 5hmC generation and depletion, and 5fC/5caC generation after grouping CpG sites according to base identity at the -1 position (top panels) or +2 position (bottom panels), reproducing the sequence context-specific trends seen in the titration assays. Data are plotted as aggregate data grouped by base identity (mean ± SD). Dashed lines are to guide visualization only.

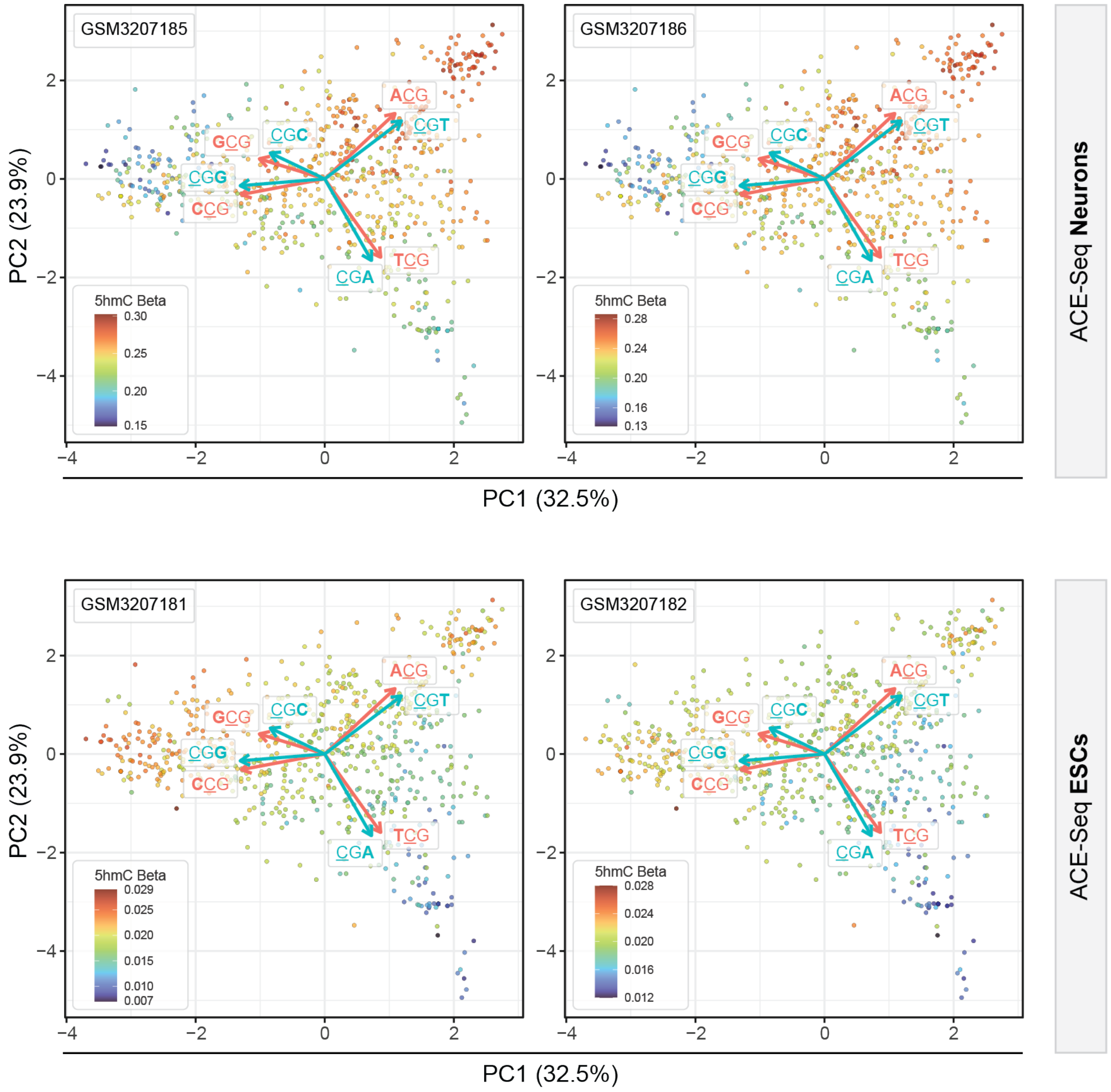

Figure S12. 5hmC accumulation tracks CpG flanking-base composition at transcription factor binding sites *in vivo*. Principal component analysis of mouse transcription factors binding sites positioned by the base composition at the -1 and +2 positions flanking the CpGs within their predicted binding sites. Each point is one transcription factor binding site; arrows show the loadings of the flanking-base features, with the axes labels colored by position (-1, red; +2, blue) and labeled by sequence context. Points are colored by the aggregate 5hmC level at CpGs overlapping each transcription factor binding site. Top row, mouse neurons; bottom row, mouse embryonic stem cells.

		EC50$_1$, μM				EC50$_2$, μM			
**4-mer**	**Compar.**	**4-mer**	**Comparison**	**Rel. eff. 4-mer**	**P-value**	**4-mer**	**Comparison**	**Rel. eff. 4-mer**	**P-value**
ACGA	ACGB	0.014 ± 0.005	0.02 ± 0.01	0.6	2E-04	0.06 ± 0.02	0.10 ± 0.04	0.6	1E-06
ACGC	ACGD	0.03 ± 0.02	0.016 ± 0.008	-0.8	1E-03	0.13 ± 0.04	0.07 ± 0.02	-0.8	4E-08
ACGG	ACGH	0.018 ± 0.009	0.02 ± 0.01	0.1	5E-01	0.08 ± 0.03	0.09 ± 0.04	0.2	2E-01
ACGT	ACGV	0.02 ± 0.01	0.02 ± 0.01	0.4	1E-01	0.07 ± 0.02	0.09 ± 0.04	0.4	1E-03
CCGA	CCGB	0.04 ± 0.02	0.06 ± 0.03	0.6	1E-05	0.19 ± 0.06	0.3 ± 0.1	0.5	5E-05
CCGC	CCGD	0.08 ± 0.04	0.05 ± 0.02	-0.6	7E-05	0.3 ± 0.2	0.21 ± 0.06	-0.7	1E-05
CCGG	CCGH	0.06 ± 0.02	0.06 ± 0.03	0.0	8E-01	0.24 ± 0.07	0.3 ± 0.2	0.2	2E-01
CCGT	CCGV	0.05 ± 0.02	0.06 ± 0.03	0.4	1E-03	0.19 ± 0.04	0.3 ± 0.2	0.5	7E-06
GCGA	GCGB	0.05 ± 0.02	0.07 ± 0.03	0.4	4E-04	0.3 ± 0.2	0.4 ± 0.3	0.4	1E-02
GCGC	GCGD	0.08 ± 0.03	0.05 ± 0.02	-0.6	4E-05	0.6 ± 0.3	0.3 ± 0.2	-0.9	5E-05
GCGG	GCGH	0.06 ± 0.02	0.06 ± 0.03	0.2	7E-02	0.3 ± 0.2	0.4 ± 0.3	0.3	4E-02
GCGT	GCGV	0.06 ± 0.02	0.06 ± 0.03	0.1	4E-01	0.3 ± 0.1	0.4 ± 0.3	0.4	7E-03
TCGA	TCGB	0.04 ± 0.02	0.06 ± 0.02	0.7	4E-07	0.16 ± 0.07	0.3 ± 0.1	0.7	5E-07
TCGC	TCGD	0.07 ± 0.02	0.05 ± 0.02	-0.6	2E-06	0.3 ± 0.1	0.19 ± 0.08	-0.8	5E-08
TCGG	TCGH	0.06 ± 0.02	0.05 ± 0.02	-0.1	3E-01	0.25 ± 0.08	0.2 ± 0.1	-0.1	5E-01
TCGT	TCGV	0.05 ± 0.01	0.06 ± 0.03	0.3	1E-02	0.18 ± 0.04	0.3 ± 0.1	0.5	1E-06

Supplementary Table 1. Comparative analysis of the 5hmC generation (EC50$_1$) and depletion (EC50$_2$) for CpG sites in different "4-mer" sequence contexts. Data from the same high-throughput TET assay utilizing context-diverse methylated substrate grouped into "4-mer" contexts by stratifying first by position -1 and then by position +2. For each 4-mer context, EC50$_1$ and EC50$_2$ values (reported as mean ± SD) were determined both for CpGs in the 4-mer itself ("4-mer") and for all other CpG sites sharing a common -1 position ("Comparison"). To facilitate comparison, the 5hmC generation or depletion efficiency of CpGs in each 4-mer relative to that of CpGs in the corresponding Comparison group ("Rel. eff. 4-mer") was calculated by a $\log_2$ transformation of the fold-change difference obtained by dividing the EC50 value of the Comparison group by the EC50 value of the associated 4-mer group. Positive and negative values reflect a relatively higher or lower efficiency, respectively, of 5hmC generation or depletion at a given 4-mer relative to all other CpGs sharing the same -1 position identity. P-values from Welch's t test reflect whether differences in the distribution of values in the two groups are statistically significant.

Aggregate (global) fitting			
	**$EC50_1$, µM**	**$EC50_2$, µM**	**Reactivity gap**
All CpGs	0.18 ± 0.01	0.43 ± 0.02	2.4 ± 0.2

Per-CpG fitting			
	**$EC50_1$, µM**	**$EC50_2$, µM**	**Average reactivity gap**
All CpGs	0.15 ± 0.05	0.5 ± 0.2	2.9 ± 0.7
**A**CG	0.09 ± 0.02	0.22 ± 0.07	2.5 ± 0.3
**C**CG	0.17 ± 0.04	0.5 ± 0.2	2.7 ± 0.5
**G**CG	0.18 ± 0.05	0.7 ± 0.3	3.7 ± 0.8
**T**CG	0.16 ± 0.03	0.5 ± 0.2	2.8 ± 0.5
CG**A**	0.13 ± 0.04	0.4 ± 0.2	2.6 ± 0.6
CG**C**	0.18 ± 0.06	0.6 ± 0.3	3.4 ± 0.8
CG**G**	0.15 ± 0.04	0.4 ± 0.2	2.9 ± 0.6
CG**T**	0.14 ± 0.04	0.4 ± 0.2	2.8 ± 0.6
	0.16 ± 0.05	0.5 ± 0.3	2.9 ± 0.8
All KvDMR CpGs			
**A**CG (KvDMR)	0.09 ± 0.02	0.23 ± 0.07	2.5 ± 0.3
**C**CG (KvDMR)	0.17 ± 0.03	0.4 ± 0.2	2.6 ± 0.5
**G**CG (KvDMR)	0.18 ± 0.05	0.8 ± 0.3	4.0 ± 0.7
**T**CG (KvDMR)	0.17 ± 0.02	0.5 ± 0.1	2.8 ± 0.4
CG**A** (KvDMR)	0.13 ± 0.04	0.3 ± 0.1	2.6 ± 0.5
CG**C** (KvDMR)	0.19 ± 0.06	0.7 ± 0.4	3.6 ± 0.9
CG**G** (KvDMR)	0.16 ± 0.04	0.5 ± 0.2	2.9 ± 0.6
CG**T** (KvDMR)	0.15 ± 0.04	0.4 ± 0.2	2.8 ± 0.7
	0.15 ± 0.05	0.5 ± 0.2	3.0 ± 0.7
All overlap CpGs			
**A**CG (overlap)	0.09 ± 0.02	0.2 ± 0.1	2.5 ± 0.3
**C**CG (overlap)	0.17 ± 0.04	0.5 ± 0.2	2.7 ± 0.4
**G**CG (overlap)	0.17 ± 0.04	0.6 ± 0.3	3.6 ± 0.8
**T**CG (overlap)	0.16 ± 0.04	0.5 ± 0.2	2.8 ± 0.5
CG**A** (overlap)	0.13 ± 0.04	0.4 ± 0.2	2.7 ± 0.6
CG**C** (overlap)	0.18 ± 0.06	0.6 ± 0.3	3.3 ± 0.8
CG**G** (overlap)	0.15 ± 0.04	0.4 ± 0.2	2.9 ± 0.7
CG**T** (overlap)	0.14 ± 0.04	0.4 ± 0.2	2.8 ± 0.6

Supplementary Table 2. TET reactivity parameters extracted from curve fits utilizing the KvDMR-containing methylated substrate. Values shown for $EC50_1$ and $EC50_2$ are mean ± SE (aggregate fitting) or mean ± SD (per-CpG fitting). Reactivity gap is the quotient obtained by dividing $EC50_2$ by $EC50_1$ for each CpG, shown as the mean ± SD across all CpG sites in a group, and represents the degree to which 5mC is preferred over 5hmC by TET as a substrate (higher values indicate greater reactivity to 5mC than to 5hmC). For per-CpG fitting, values are shown as averages integrating first across all CpG sites in the pNE031 substrate, then across only CpG sites within the sequence corresponding to the KvDMR locus ("KvDMR CpGs"), and finally across all CpG sites that are present in both this substrate and the context-diverse methylated substrate ("overlap CpGs").
